# A low-swelling hydrogel as a multirole sealant for efficient dural defect sealing and prevention of postoperative adhesion

**DOI:** 10.1093/nsr/nwae160

**Published:** 2024-04-30

**Authors:** Xueliang Cheng, Zhen Zhang, Hui Ren, Zheng Zou, Yu Zhang, Yang Qu, Xuesi Chen, Jianwu Zhao, Chaoliang He

**Affiliations:** CAS Key Laboratory of Polymer Ecomaterials, Changchun Institute of Applied Chemistry, Chinese Academy of Sciences, Changchun 130022, China; Department of Orthopedics, The Second Norman Bethune Hospital of Jilin University, Changchun 130014, China; CAS Key Laboratory of Polymer Ecomaterials, Changchun Institute of Applied Chemistry, Chinese Academy of Sciences, Changchun 130022, China; CAS Key Laboratory of Polymer Ecomaterials, Changchun Institute of Applied Chemistry, Chinese Academy of Sciences, Changchun 130022, China; School of Applied Chemistry and Engineering, University of Science and Technology of China, Hefei 230026, China; CAS Key Laboratory of Polymer Ecomaterials, Changchun Institute of Applied Chemistry, Chinese Academy of Sciences, Changchun 130022, China; School of Applied Chemistry and Engineering, University of Science and Technology of China, Hefei 230026, China; CAS Key Laboratory of Polymer Ecomaterials, Changchun Institute of Applied Chemistry, Chinese Academy of Sciences, Changchun 130022, China; Department of Orthopedics, The Second Norman Bethune Hospital of Jilin University, Changchun 130014, China; CAS Key Laboratory of Polymer Ecomaterials, Changchun Institute of Applied Chemistry, Chinese Academy of Sciences, Changchun 130022, China; School of Applied Chemistry and Engineering, University of Science and Technology of China, Hefei 230026, China; Department of Orthopedics, The Second Norman Bethune Hospital of Jilin University, Changchun 130014, China; CAS Key Laboratory of Polymer Ecomaterials, Changchun Institute of Applied Chemistry, Chinese Academy of Sciences, Changchun 130022, China; School of Applied Chemistry and Engineering, University of Science and Technology of China, Hefei 230026, China

**Keywords:** hydrogel sealant, watertight closure, low swelling, *o*-phthalaldehyde chemistry, dural sealing

## Abstract

Dural defects and subsequent complications, including cerebrospinal fluid (CSF) leakage, are common in both spine surgery and neurosurgery, and existing clinical treatments are still unsatisfactory. In this study, a tissue-adhesive and low-swelling hydrogel sealant comprising gelatin and *o*-phthalaldehyde (OPA)-terminated 4-armed poly(ethylene glycol) (4aPEG-OPA) is developed via the OPA/amine condensation reaction. The hydrogel shows an adhesive strength of 79.9 ± 12.0 kPa on porcine casing and a burst pressure of 208.0 ± 38.0 cmH_2_O. The hydrogel exhibits a low swelling ratio at physiological conditions, avoiding nerve compression in the limited spinal and intracranial spaces. In rat and rabbit models of lumbar and cerebral dural defects, the 4aPEG-OPA/gelatin hydrogel achieves excellent performance in dural defect sealing and preventing CSF leakage. Moreover, local inflammation, epidural fibrosis and postoperative adhesion in the defect areas are markedly reduced. Thus, these findings establish the strong potential of the hydrogel sealant for the effective watertight closure of dural defects.

## Teaser

A tissue-adhesive and low-swelling hydrogel sealant is developed via the o-phthalaldehyde/amine condensation reaction for efficient sutureless closure and watertight sealing of dural defects, as well as prevention of post-operative adhesion.

## INTRODUCTION

As the outermost layer of the meninges, the dura mater is a fibrous membrane of connective tissue that covers the spinal cord and the brain [1]. Many neurosurgeries and spinal surgeries involving access to the underlying nervous tissues create defects in the dura mater, further resulting in cerebrospinal fluid (CSF) leakage [2]. CSF leakage can lead to severe complications, including postural headache, pseudodural cyst, dural fistula, cerebral/spinal hernia, meningitis, intracranial hemorrhage, epidural fibrosis, postoperative adhesion, etc. [3–5]. These complications further cause issues such as prolonged hospital stays, increased medical expenses, and consequences related to long-term bed rest (accumulated pneumonia, bedsore, lower extremity deep venous thrombosis, and urinary tract infection). Therefore, rapid and watertight closure of dural damage is imperative in neurosurgical operations.

To achieve watertight closure, clinical treatment mainly focuses on suturing the defect area [6,7]. However, this method has several disadvantages. Sutures are time-consuming and technically difficult, especially when the defects are in inaccessible areas (such as the axilla and nerve root sleeve) [8]. Suturing causes damage to the dura mater, and needle holes pose additional challenges to achieving watertight closure [9]. In addition, suturing may lead to secondary stenosis of the related nerve or spinal cord and change the low pressure in the dural defect area into high pressure, resulting in continuous CSF leakage [10]. In recent years, it has been reported that dural sealants are more desirable for better long-term clinical outcomes of dural repair than sutures or nontreatment in some cases with relatively small defects (<3 mm) [11].

In recent years, the development of tissue adhesives and sealants for sutureless wound closure, hemostasis and leakage-proof sealing has received widespread attention due to their advantages including convenience, decreased damage to tissues, and suitability for emergency situations and complicated locations and wounds [12–20]. In particular, hydrogel-based adhesives and sealants consisting of cross-linked hydrophilic polymer chains dispersed in aqueous media have attracted intense interest due to their good biocompatibility, favorable adaptability to wounds with complicated shapes, and tunable physicochemical and biological functions [21–27]. For dural sealing, the current clinical products mainly include fibrin glue and poly(ethylene glycol) (PEG)-based hydrogel sealants. Fibrin glue, mainly consisting of animal-derived fibrinogen and thrombin, is a widely used sealant in clinical applications and forms a hydrogel via the blood coagulation mechanism [28]. Although fibrin glue has good biodegradability, its application is limited by its weak mechanical properties and risk of virus transmission [29,30]. DuraSeal, which is a hydrogel sealant consisting of trilysine and 4-armed PEG with *N*-hydroxysuccinimide (NHS) ester end groups (4aPEG-NHS), was developed in order to achieve watertight closure in dural repair. The components of the sealant form a cross-linking network through amidation between NHS ester groups of 4aPEG-NHS and amine groups in trilysine. Additionally, the sealant achieves covalent tissue adhesion by coupling the NHS ester groups of 4aPEG-NHS with the amine groups on the tissue surface. However, a major problem with DuraSeal is excessive swelling under physiological conditions, which is extremely detrimental and sometimes fatal in the limited spinal and intracranial spaces [31–34]. Additionally, the adhesion mechanism based on the NHS ester/amine coupling reaction may lead to limited operation time and compromised tissue adhesion performance due to the hydrolytic instability of the NHS ester after dissolution in aqueous medium [35].

In recent years, researchers have focused on the development of advanced dural sealants to overcome the limitations of current products [36–41]. Zhao and co-authors reported an injectable hydrogel with rapid adhesion and anti-swelling properties [42]. The precursor solution composed of methacrylated hyaluronic acid, acrylated nano-micelles gelator, and acrylic acid NHS ester could be injected through a needle and form a hydrogel upon UV illumination. The hydrogel displayed non-expansion of volume during degradation owing to the thermo-sensitive shrinkable micelle cores. However, the presence of an initiator and UV radiation can pose potential safety issues. Wu and co-workers applied alginate-polyacrylamide hydrogel with high toughness and maximum stress for intraoperative sealing of the dura mater [43]. The hydrogel could adhere to the porcine dura under pressure up to 100 mmHg on Yorkshire pigs. Nevertheless, this kind of preformed hydrogel may not be convenient to use in difficult-to-access tissue sites or in minimally invasive surgery. Overall, there is still great demand to prepare a new type of dural sealant with good biocompatibility, superior sealing performance, and a low swelling ratio.

As a product of the partial degradation of collagen, gelatin is a promising candidate for preparing dural sealants, owing to its low immunogenicity, biodegradability, excellent biocompatibility and low cost [44]. In addition, the functional groups of gelatin, such as amino groups, can be used for cross-linking or further modification [45,46]. PEG has good hydrophilicity and biocompatibility and has been approved by the FDA for clinical use. Moreover, the end groups of PEG can be easily modified with functional groups. In our recent study, it was discovered that the reaction of *o*-phthalaldehyde (OPA) with primary amino groups could be used for the rapid formation of hydrogels [47]. During the cross-linking process, the two adjacent formyl groups of OPA provide an intramolecular trap for amino groups, leading to the rapid formation of a stable heterocycle product, phthalimidine, as the cross-link. The OPA/amine condensation reaction proceeds spontaneously and chemoselectively under physiological conditions, with water as the only byproduct [48,49]. Moreover, the potential coupling reaction between the OPA groups and amino groups at the tissue surface can be utilized to achieve rapid and strong adhesion between the hydrogels and tissues, which provides a strategy for the design of a new type of tissue sealant.

**Figure 1.**
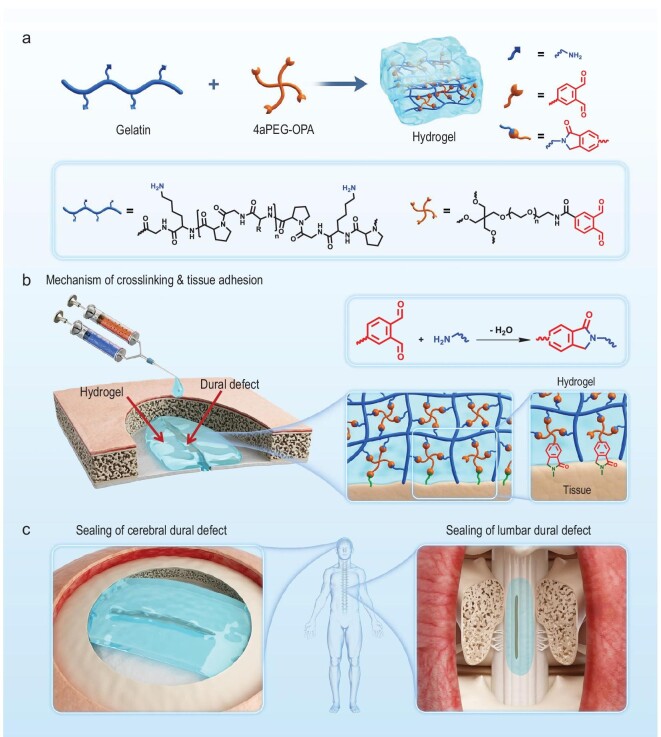
(a) Fabrication of hydrogel sealants by mixing gelatin with 4aPEG-OPA. (b) Schematic mechanisms for stable cross-linking in bulk hydrogel and firm adhesion between the hydrogel and tissue surface. (c) Application of hydrogel sealants in sealing and repairing defects in cerebral and lumbar dura mater.

Therefore, in this work, an injectable, low-swelling hydrogel sealant was developed based on OPA chemistry for dural sealing and repair (Fig. 1). The hydrogel was formed by simply mixing gelatin and OPA-terminated 4-armed PEG (4aPEG-OPA). The OPA groups of 4aPEG-OPA could react with amine groups on gelatin to form phthalimidine linkages, resulting in a stable cross-linking network with low-swelling properties. Meanwhile, the OPA groups could couple with amine groups on the tissue surface, which contributed to firm tissue adhesion of the hydrogel and watertight sealing of dural defects. The gelation behaviors, mechanical strength, degradation profiles and biocompatibility of the hydrogel were investigated in detail. In rat and rabbit models of lumbar and cerebral dural defects, the sealing and repair performance of the 4aPEG-OPA/gelatin hydrogel were evaluated *in vivo* and compared with the performance of a commercially available fibrin glue and a hydrogel composed of 4aPEG-OPA and cystamine-modified 4-armed PEG (4aPEG-SSNH_2_).

## RESULTS AND DISCUSSION

### Preparation and characterization of the hydrogel sealants

4aPEG-OPA was synthesized according to our previously reported method (Fig. S1) [47]. The ^1^H NMR spectrum of the product showed two characteristic peaks of the aldehyde groups of the OPA moiety at 10.52 ppm and 10.61 ppm, indicating the successful synthesis of 4aPEG-OPA (Fig. S2). Additionally, 4aPEG-SSNH_2_ was synthesized via a two-step reaction composed of the reaction between 4-armed PEG and *p*-nitrophenyl chloroformate (NPC), followed by the reaction of NPC-conjugated 4-armed PEG with cystamine (Fig. S3).

**Figure 2.**
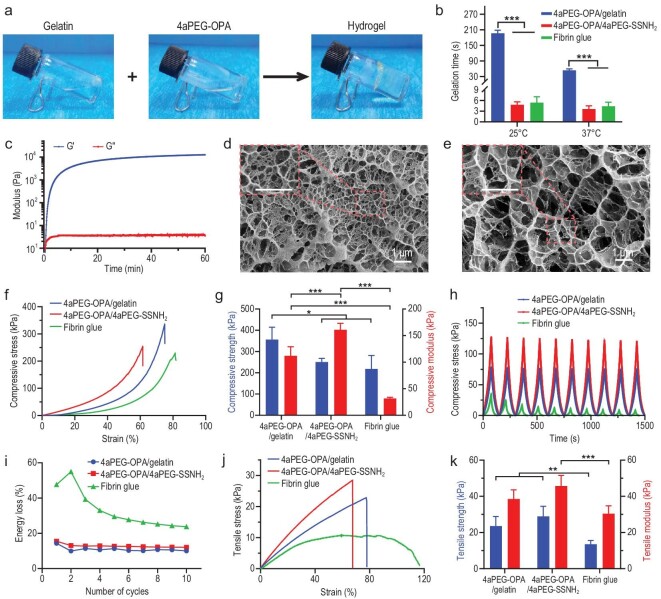
(a) Images showing the formation of hydrogel by mixing gelatin and 4aPEG-OPA in PBS. (b) Gelation time of 4aPEG-OPA/gelatin, 4aPEG-OPA/4aPEG-SSNH_2_, and fibrin glue at 25°C and 37°C (mean ± SD, *n* = 5). (c) Time-sweep rheological test of the mixture of 4aPEG-OPA and gelatin at 37°C. (d) Cryo-SEM image of the 4aPEG-OPA/gelatin hydrogel. The scale bars represent 1 μm. (e) Cryo-SEM image of the 4aPEG-OPA/4aPEG-SSNH_2_ hydrogel. The scale bars represent 1 μm. (f) Representative compressive stress‒strain curves of different hydrogels. (g) Compressive strengths and moduli (mean ± SD, *n* = 5). (h) Cyclic compressive curves of different hydrogels. (i) Energy loss calculated from cyclic compressive tests. (j) Representative tensile stress‒strain curves of different hydrogels. (k) Tensile strengths and moduli (mean ± SD, *n* = 7). *P < 0.05, **P < 0.01, ***P < 0.001.

The hydrogels were prepared by simply mixing 4aPEG-OPA and gelatin or 4aPEG-SSNH_2_ (1/1 (w/w), 10% (w/v)) in phosphate-buffered saline (PBS) (Fig. 2a). The gelation mechanism is mainly based on the condensation reaction between the OPA groups of 4aPEG-OPA and the amino groups of gelatin or 4aPEG-SSNH_2_, with the formation of stable phthalimidine linkages [47]. The ^1^H NMR spectrum showed that the characteristic peaks of OPA groups of 4aPEG-OPA had almost disappeared after mixing with gelatin (Fig. S4). It is noteworthy that, for 4aPEG-OPA/gelatin hydrogel, the hydrogen bonds between gelatin/gelatin and gelatin/PEG chains could provide the hydrogel with additional multiple cross-links. The FTIR spectrum of 4aPEG-OPA/gelatin hydrogel displayed characteristic N-H, C=O, and O−C−O stretching vibration peaks at 3300, 1643, and 1088 cm^−1^ (Fig. S5). In contrast, for 4aPEG-OPA/4aPEG-SSNH_2_ hydrogel, the peaks of N−H, C=O, and O−C−O stretching vibration were found at 3346, 1658, and 1097 cm^−1^. The redshift of these peaks in 4aPEG-OPA/gelatin hydrogel suggested that hydrogen bonding was formed between the polymer chains [50,51].

The gelation time at room temperature (25°C) and at physiological temperature (37°C) was tested by the tube inversion method. As shown in Fig. 2b, the gelation time of the 4aPEG-OPA/gelatin hydrogel was ∼196.4 ± 11.8 s at 25°C and 52.4 ± 5.6 s at 37°C. Increasing the weight ratio of 4aPEG-OPA to gelatin could slightly decrease the gelation time (Fig. S6). The 4aPEG-OPA/4aPEG-SSNH_2_ hydrogel and fibrin glue showed quite short gelation times (5–10 s). Notably, the gelation time of the 4aPEG-OPA/gelatin hydrogel is preferred for operation by clinicians because inappropriate gelation time can lead to clogging of the application device or insufficient sealant coverage and subsequently compromising sealing performance.

The evolution over time of the storage modulus (G′) and loss modulus (Gʺ) of the 4aPEG-OPA/gelatin hydrogel during the cross-linking process was measured by a rheometer at 37°C. As shown in Fig. 2c, the G′ value increased rapidly and tended to reach a plateau of 12.6 kPa after 60 min, suggesting the rapid formation of a viscoelastic hydrogel. The hydrogels with different weight ratios of 4aPEG-OPA to gelatin showed comparable gelation kinetics and plateau G′ (Fig. S7). By contrast, increasing the polymer concentration could considerably increase the plateau G′ of the hydrogels.

The microscopic morphology of the hydrogels was observed by cryo-scanning electron microscopy (cryo-SEM). As shown in Fig. 2d and e, the cryo-SEM image revealed homogeneous porous network structures of the 4aPEG-OPA/gelatin and 4aPEG-OPA/4aPEG-SSNH_2_ hydrogels. The pore sizes of the hydrogels ranged from tens to hundreds of nanometers. The results suggested that the hydrogels may be an effective physical barrier to prevent postoperative adhesion since the pore sizes were smaller than the size of fibroblasts (≥2–3 μm) [52].

The mechanical properties of the hydrogel sealants were evaluated by using a universal testing machine. As shown in Fig. 2f and g, the compressive moduli of the 4aPEG-OPA/gelatin and 4aPEG-OPA/4aPEG-SSNH_2_ hydrogels were 112.0 ± 17.2 kPa and 161.0 ± 12.4 kPa, respectively, which were much higher than that of fibrin glue (32.0 ± 2.1 kPa). The compressive strength at failure of the 4aPEG-OPA/gelatin hydrogel was 356.3 ± 58.7 kPa, which was higher than that of the 4aPEG-OPA/4aPEG-SSNH_2_ hydrogel. The weight ratio of the precursor polymers could affect the compressive properties of the resultant hydrogels. The 4aPEG-OPA/gelatin hydrogel displayed relatively high compressive strengths with a 4aPEG-OPA to gelatin weight ratio of 1:1 (Fig. S8). In addition, cyclic compressive tests were carried out to assess the fatigue resistance of the hydrogel sealants. The results showed that the 4aPEG-OPA/gelatin hydrogel could recover to its initial state after compression (Fig. 2h and Fig. S9). The energy loss calculated from the stress-strain curve also suggested that the 4aPEG-OPA/gelatin hydrogel had better anti-fatigue performance compared with fibrin glue (Fig. 2i). The tensile modulus and tensile strength at failure of the 4aPEG-OPA/gelatin hydrogel were 38.5 ± 5.0 kPa and 24.4 ± 5.1 kPa (Fig. 2j, k and Fig. S10), respectively, which were significantly higher than those of fibrin glue. Overall, the compressive and tensile tests demonstrated the superior mechanical performance of the 4aPEG-OPA/gelatin hydrogel compared with fibrin glue.

### Burst pressure and sealing performance of the hydrogel sealants

**Figure 3.**
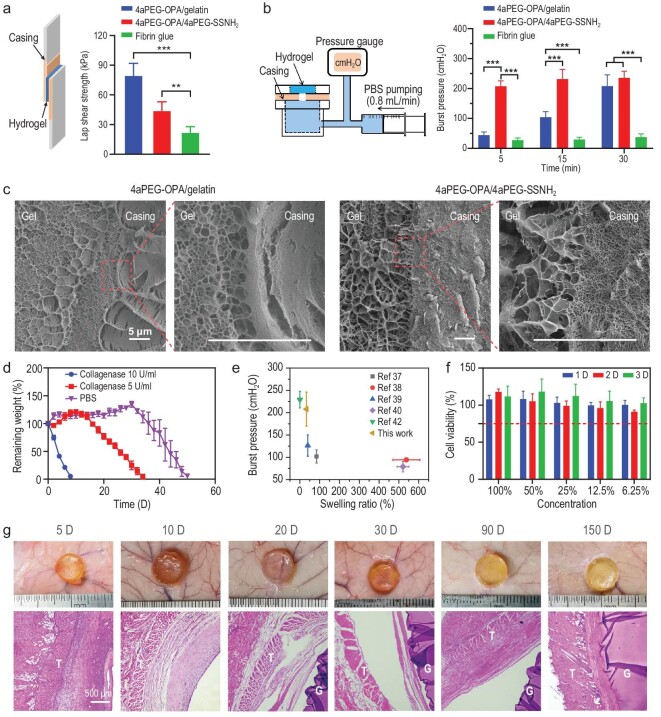
(a) Schematic diagram of the lap shear test and lap shear strengths of fibrin glue, 4aPEG-OPA/4aPEG-SSNH_2_ and 4aPEG-OPA/gelatin (mean ± SD, *n* = 6). (b) Schematic diagram of the *in vitro* burst pressure test and *in vitro* burst pressure of different hydrogels at different time points (mean ± SD, *n* = 6). (c) Cryo-SEM images of the interface between the hydrogel and porcine casing. The scale bars represent 5 μm. (d) Degradation profiles of the 4aPEG-OPA/gelatin hydrogel in blank PBS and PBS containing type IV collagenase (mean ± SD, *n* = 3). (e) Comparison of 4aPEG-OPA/gelatin and other reported hydrogels for dural sealing in terms of swelling ratios and burst pressures. (f) Cell viability of NIH 3T3 cells incubated with the 4aPEG-OPA/gelatin extracts for 1 D, 2 D, and 3 D (mean ± SD, *n* = 5). (g) Images of the 4aPEG-OPA/gelatin hydrogels after subcutaneous implantation in rats and H&E staining images of surrounding skin tissues at different time points. T indicates the location of the tissue, and G indicates the location of the hydrogel. **P < 0.01, ***P < 0.001.

The adhesive strength of the hydrogel sealants on *ex vivo* tissue (porcine casing) was determined by lap shear test. As shown in Fig. 3a and Fig. S11, the adhesive strength of the 4aPEG-OPA/gelatin hydrogel was 79.9 ± 12.0 kPa, which was markedly higher than that of fibrin glue (22.4 ± 5.5 kPa). Although the 4aPEG-OPA/4aPEG-SSNH_2_ hydrogel exhibited comparable compressive and tensile strength, the tissue adhesive strength was relatively weak (44.4 ± 8.6 kPa) compared with that of the 4aPEG-OPA/gelatin hydrogel. This may be ascribed to the fact that the very short gelation time (∼5 s) of the 4aPEG-OPA/4aPEG-SSNH_2_ hydrogel makes it difficult to apply the hydrogel to uniformly coat the overlap area of tissues.

The *in vitro* sealing performance of the hydrogel sealants was evaluated by burst pressure test (Fig. 3b). At 5 min after applying the hydrogel, the burst pressure of the 4aPEG-OPA/gelatin hydrogel was 44.3 ± 10.5 cmH_2_O, which was higher than that of fibrin glue (27.6 ± 7.3 cmH_2_O). Moreover, the burst pressure of the 4aPEG-OPA/gelatin hydrogel was enhanced markedly with increasing incubation time, whereas the burst pressure of fibrin glue showed no obvious change. For instance, after incubating for 30 min, the burst pressure of the 4aPEG-OPA/gelatin hydrogel was increased to 208.0 ± 38.0 cmH_2_O, which was comparable to that of the 4aPEG-OPA/4aPEG-SSNH_2_ hydrogel (235.6 ± 22.3 cmH_2_O). The interface between the hydrogel and tissue was observed by using cryo-SEM. As shown in Fig. 3c, a tight interface was formed between porcine casing and 4aPEG-OPA/gelatin or 4aPEG-OPA/4aPEG-SSNH_2_ hydrogel.

The tissue adhesion and sealing performances of the 4aPEG-OPA/gelatin hydrogel is attributed to the coupling reaction between the OPA groups and the amino groups on the tissue surface (Fig. 1). Aside from porcine casing, the 4aPEG-OPA/gelatin hydrogel also demonstrated adhesion properties to various tissues, including skin, organs, and dura mater. The 180° peel test on porcine skin showed that the interfacial toughness of 4aPEG-OPA/gelatin hydrogel was 62.7 ± 3.3 J/m^2^ (Fig. S12). After applying into the incision of rabbit heart, liver, spleen, and kidney, the 4aPEG-OPA/gelatin hydrogel could effectively adhere to the tissues and achieve incision closure (Fig. S13). Besides, cryo-SEM images showed that the 4aPEG-OPA/gelatin hydrogel tightly adhered to the porcine dura mater, resulting in a closely connected hydrogel-tissue interface (Fig. S14).

In this study, we tested the *in vitro* burst pressure after incubating the samples for 5 min, 15 min, and 30 min. The three time points were determined according to the clinical usage scenario. At the early stage, the sealant is required to quickly seal the dural defect and prevent CSF leakage. At 5 min after treatment with the sealant, the burst pressure of the 4aPEG-OPA/gelatin hydrogel exceeded the normal CSF pressure (8–18 cmH_2_O) in the lateral decubitus position [53]. After the operation is finished (15–30 min), the surgical position of patients often needs to be changed. The CSF pressure fluctuations caused by postural changes or other factors (sneezing, vomiting, coughing, etc.) are the main reasons for the failure of materials to seal dural defects [54,55]. For instance, the lumbar CSF pressure can exceed 100 cmH_2_O when a normal adult is standing [56]. The burst pressures of both the 4aPEG-OPA/gelatin and 4aPEG-OPA/4aPEG-SSNH_2_ hydrogels developed in this study exceeded 200 cmH_2_O at 30 min post-treatment, surpassing the fluctuation level of postoperative CSF pressure.

To further demonstrate the superior sealing performance of the hydrogel sealants *in vivo*, we conducted a defect sealing experiment in a rabbit model of cerebral dural defect (Fig. S15). After applying the sealants for 5 min, the infusion of PBS was started at a rate of 0.8 mL/min, which is twice the normal flow rate of CSF [57]. With the infusion of PBS, liquid leakage occurred first on the defect treated with fibrin glue compared to the 4aPEG-OPA/gelatin and 4aPEG-OPA/4aPEG-SSNH_2_ hydrogels (Fig. S15). This confirmed that both the 4aPEG-OPA/gelatin and 4aPEG-OPA/4aPEG-SSNH_2_ hydrogels were more effective in sealing dural defects.

Considering that CSF leakage is continuous in actual clinical situations, we also explored the instant sealing effect of the hydrogel sealants. In this experiment, PBS was pumped at a rate of 0.8 mL/min, and the pressure was maintained at ∼10 cmH_2_O to simulate CSF circulation [57]. The hydrogels were added while the defect was actively leaking. As shown in Fig. S16, both the 4aPEG-OPA/gelatin and 4aPEG-OPA/4aPEG-SSNH_2_ hydrogels could effectively seal the leakage, while obvious leakage was observed in the defect treated with fibrin glue.

### Degradation and biocompatibility of the hydrogel sealants

The degradability of a sealant is highly important, as it circumvents the issue of postoperative removal. Therefore, we investigated the *in vitro* degradation of the hydrogel sealants. As shown in Fig. 3d, in blank PBS, the 4aPEG-OPA/gelatin hydrogels exhibited slight swelling in the first 30 days due to the gradual decrease in the cross-linking density resulting from hydrolysis of the gelatin backbone [58]. After 30 days, the hydrogels showed an obvious mass loss and degraded completely within 50 days, owing to the breakage of the cross-linking network. The degradation periods could be reduced to 20 days and 9 days by adding 5 U/mL and 10 U/mL collagenase, respectively, which were attributed to the accelerated cleavage of the gelatin backbone in the presence of collagenase.

In contrast, the 4aPEG-OPA/4aPEG-SSNH_2_ hydrogels showed degradation behavior dependent on reductive molecules, such as glutathione (GSH). As shown in Fig. S17, in blank PBS, no obvious degradation of the 4aPEG-OPA/4aPEG-SSNH_2_ hydrogels was found within 50 days, whereas the hydrogels were degraded within 20 days in the presence of 10 mM GSH, caused by the breakage of disulfide linkages.

Commercially available dural sealants, such as DuraSeal, usually exhibit excessive swelling (50%–400%) [59–62]. When used in the spinal canal, the swelling of implanted biomaterials can compress the spinal cord and lead to severe complications [63]. Recently, efforts have been made on the development of hydrogel-based dural sealants. Notably, to reduce the swelling ratios of hydrogels, the stability of hydrogel networks was enhanced by introduction of hydrophobic interactions between hydrophobic side groups or thermo-induced micellar self-assembly [39,42]. In this work, the 4aPEG-OPA/gelatin hydrogel exhibited a low swelling ratio (33.3% ± 5.0%) and relatively high burst pressure (Fig. 3e and Table S1). The low-swelling properties of the 4aPEG-OPA/gelatin hydrogel could be attributed to the irreversible phthalimidine linkages formed between the OPA groups of 4aPEG-OPA and the amino groups of gelatin, as well as the multiple cross-links provided by the hydrogen bonds between gelatin/gelatin and gelatin/PEG chains [64]. Additionally, the ionic interactions and hydrophobic interactions within the gelatin chains may also contribute to the low swelling ratios of the 4aPEG-OPA/gelatin hydrogel.

To evaluate the *in vitro* cytotoxicity of the hydrogel sealants, NIH 3T3 cells and L929 cells were incubated with the extracts of 4aPEG-OPA/gelatin or 4aPEG-OPA/4aPEG-SSNH_2_ hydrogel. The viability of NIH 3T3 cells was higher than 85% (Fig. 3f and Fig. S18). Besides, it was observed by live-dead staining that most NIH 3T3 cells and L929 cells were viable with green fluorescence (Fig. S18). Therefore, both the 4aPEG-OPA/gelatin and 4aPEG-OPA/4aPEG-SSNH_2_ hydrogels exhibited good cytocompatibility *in vitro*.

In hemolysis assay, red blood cell suspension was incubated with the extracts of 4aPEG-OPA/gelatin or 4aPEG-OPA/4aPEG-SSNH_2_ hydrogel. As shown in Fig. S19, the supernatant incubated after 1 h and 3 h displayed a light red color similar to that of the negative control (normal saline). The hemolysis ratios were lower than 2.5%. The results confirmed the good hemocompatibility of the hydrogel sealants *in vitro*.

The histocompatibility of the hydrogel sealants was evaluated by subcutaneous implantation in rats. As shown in Fig. 3g and Fig. S20, the hydrogels could maintain their integrity for up to 150 days, and a capsule formed around the hydrogels. No tissue necrosis, edema, or hyperemia was observed throughout the experiments. The hydrogels were collected, and their weights were recorded. As shown in Fig. S21, the 4aPEG-OPA/gelatin hydrogels showed slight swelling over time, with a swelling ratio of ∼30% *in vivo* after 150 days, which was consistent with the swelling ratios *in vitro*.

The subcutaneous degradation rate of the 4aPEG-OPA/gelatin hydrogels in rats was slower than that in the *in vitro* experiments. This could be ascribed to the slower exchange of subcutaneous body fluid in rats compared to the degradation media *in vitro* [65]. In addition, the capsule formation surrounding the hydrogels further reduced body fluid exchange. Since hydrogels can exist stably in the body for a certain period of time, they can effectively form a physical barrier, which provides the possibility to prevent epidural fibrosis and undesired postoperative adhesion to epidural tissues.

The skin tissues in contact with the hydrogels and major organs were collected for histological analysis. H&E staining showed that the hydrogels induced a mild inflammatory reaction at 5 days after implantation (Fig. 3g). The inflammatory reaction subsided over time, suggesting that the hydrogel has good biocompatibility at the tissue level.

### Sealing and repair of defects in dura mater spinalis of rats

**Figure 4.**
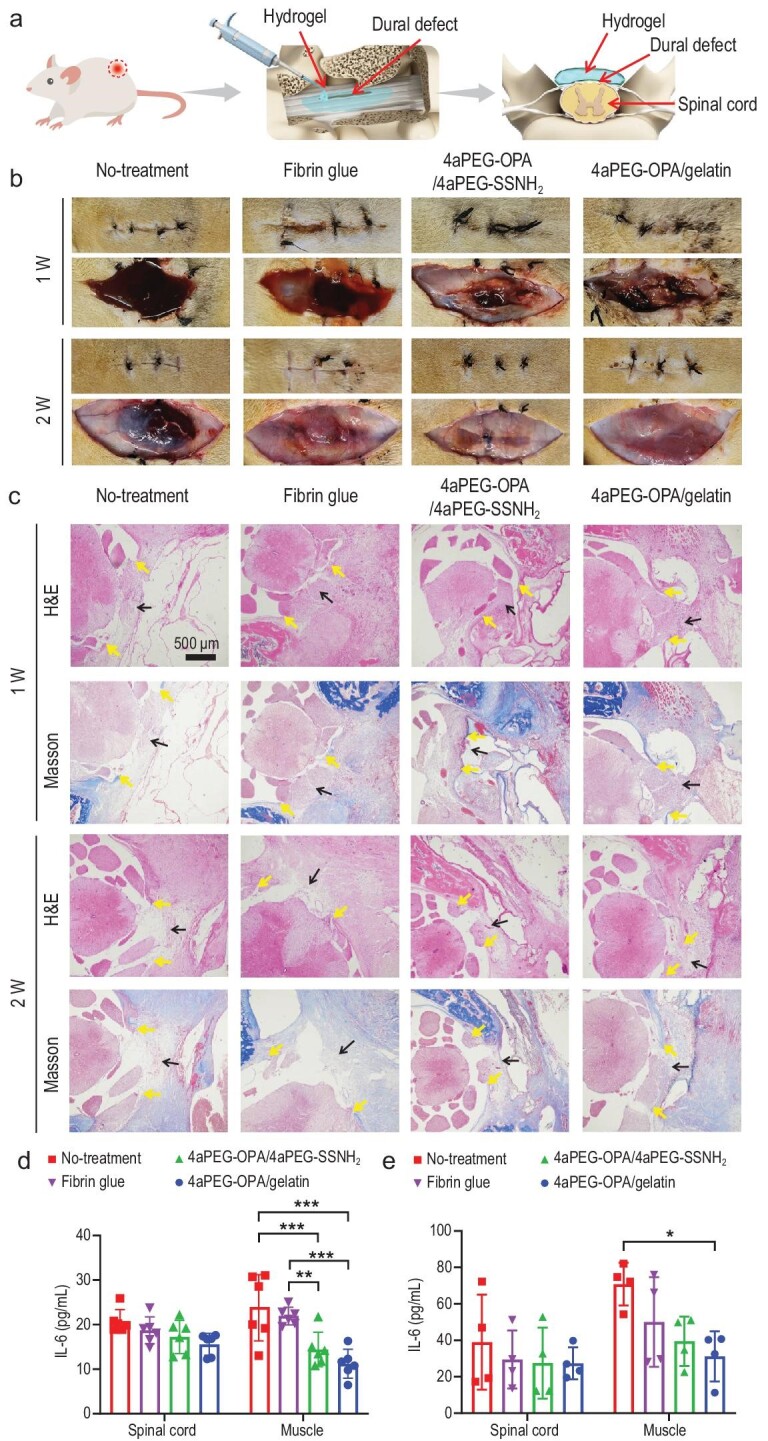
(a) Schematic diagram of the sealing and repair of defects in the dura mater spinalis of rats. (b) Visual inspection of surgical sites before and after dissecting the skin incisions for the no-treatment, fibrin glue, 4aPEG-OPA/4aPEG-SSNH_2_, and 4aPEG-OPA/gelatin groups. The upper row of images for each group represents the sutured skin layer covering the wounds, and the lower row of images for each group indicates the exposed wounds. (c) H&E and Masson's trichrome staining for different groups. Black arrows indicate the dural defect area. Yellow arrows indicate the ends of dural defects. (d) IL-6 content of spinal cord and surface muscle at 1 week (mean ± SD, *n* = 6). (e) IL-6 content of spinal cord and surface muscle at 2 weeks (mean ± SD, *n* = 4). *P < 0.05, **P < 0.01, ***P < 0.01.

To evaluate the *in vivo* sealing performance of the hydrogel sealants, a defect of ∼1 cm was created in the dura mater spinalis of rats, and the hydrogels were applied to cover the dural defect (Fig. 4a). Fibrin glue was also used according to the manufacturer's instructions for comparison, and no sealing was performed in the no-treatment group. As shown in Fig. 4b, in the no-treatment group, a subcutaneous mass ∼3–4 cm in diameter was observed at 1 week post-treatment, and a distinct subcutaneous pseudocyst formed due to the accumulation of blood and CSF at 2 weeks [6]. A smaller subcutaneous mass and less CSF leakage also existed after treatment with fibrin glue. In contrast, after treatment with the 4aPEG-OPA/gelatin and 4aPEG-OPA/4aPEG-SSNH_2_ hydrogels, no obvious hemorrhage, CSF accumulation, or pseudocyst formation was observed.

Histological assessment was carried out by H&E staining. As shown in Fig. 4c, at 1-week post-operation, the dura mater defects were clearly visible, and granulation tissue had not yet completely filled the defect areas in the no-treatment and fibrin glue groups. In the group treated with the 4aPEG-OPA/4aPEG-SSNH_2_ hydrogel, although the dural defect was sealed, the spinal cord in the defect area was compressed, likely due to the swelling of the 4aPEG-OPA/4aPEG-SSNH_2_ hydrogel [31–34]. In contrast, after treatment with 4aPEG-OPA/gelatin hydrogel for 1 week, the dura mater defect was sealed, and obvious granulation tissue could be observed filling in the dural defect area. Collagen is a major component of the extracellular matrix of the dura mater [66]. The formation of collagen fibers was further evaluated by Masson's trichrome staining. At 1 week, discontinuous blue collagen fibers could be found in the dural defect area in the 4aPEG-OPA/gelatin hydrogel group. At 2 weeks after treatment, the collagen in the dural defect areas of the 4aPEG-OPA/4aPEG-SSNH_2_ and 4aPEG-OPA/gelatin groups was noticeably denser than that in the no-treatment group and the fibrin glue group. The results confirmed that both the 4aPEG-OPA/4aPEG-SSNH_2_ and 4aPEG-OPA/gelatin hydrogels can effectively block dural defects and promote dural repair, whereas obvious spinal cord compression was observed in the 4aPEG-OPA/4aPEG-SSNH_2_ hydrogel-treated rats due to the marked hydrogel swelling.

The exuded CSF in the epidural tissues can stimulate the inflammatory response of the body and promote the secretion of the proinflammatory factor. Excessive expression of proinflammatory factors can induce excessive tissue proliferation, which may in turn cause epidural fibrosis and undesired postoperative adhesions [67,68]. We collected the spinal cord and muscle tissues of rats to analyze the inflammatory factors. At 1-week post-treatment, the IL-6 content of muscle tissues for the no-treatment group and the fibrin glue group were significantly higher than those for the 4aPEG-OPA/4aPEG-SSNH_2_ and 4aPEG-OPA/gelatin hydrogel groups (Fig. 4d and e). Moreover, at 2 weeks, the IL-6 content of muscle tissues in the no-treatment group was still significantly higher than that in the 4aPEG-OPA/gelatin hydrogel group. There was no significant difference in the contents of tumor necrosis factor α in spinal cords or muscle tissues between different groups (Fig. S22). This result confirmed that the sealing and repair of dural defects by 4aPEG-OPA/gelatin hydrogels could effectively relieve the local inflammatory response and reduce the risk of postoperative epidural fibrosis.

### Sealing and repair of defects in cerebral dura mater of rabbits

**Figure 5.**
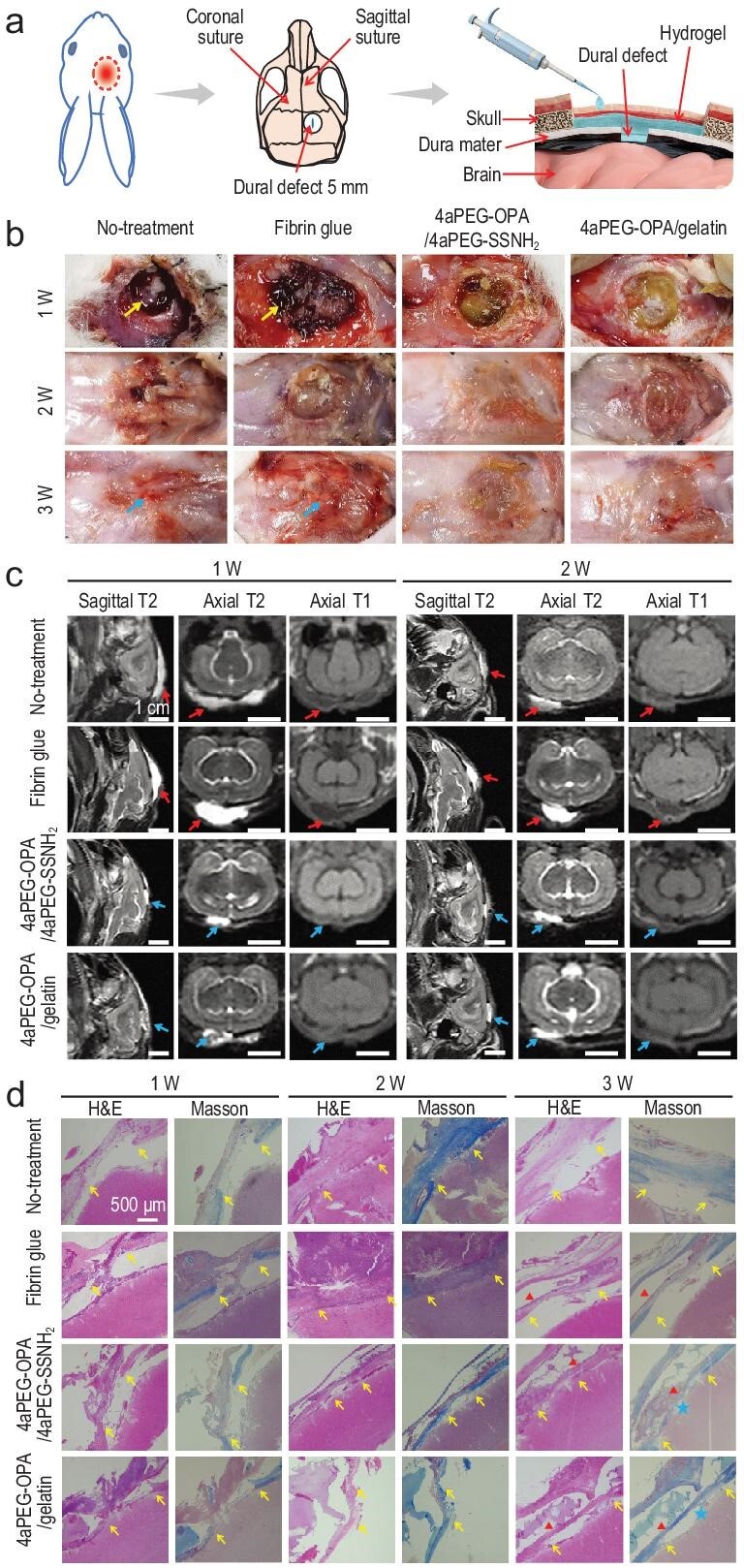
(a) Schematic diagram of the sealing and repair of defects in the cerebral dura mater of rabbits. (b) Visual inspection of surgical sites after dissecting the skin incisions for the no-treatment, fibrin glue, 4aPEG-OPA/4aPEG-SSNH_2_, and 4aPEG-OPA/gelatin groups. Yellow arrows indicate CSF exudation. Blue arrows indicate congestion after treatment. (c) The head MRI results of rabbits for different groups. Red arrows indicate accumulated cerebrospinal fluid, and blue arrows indicate residual hydrogels. (d) H&E and Masson's trichrome staining of cerebral dura mater for different groups. Yellow arrows indicate the ends of the dural defect; blue pentagrams indicate new collagen deposition; red triangles indicate residual hydrogels or subcutaneous cavities formed after the degradation of fibrin glue.

We further investigated the sealing performance of the hydrogel sealants in the dura mater cerebralis of rabbits [69,70]. A defect in the cerebral dura mater (5 mm in length) was created, and hydrogel or fibrin glue was applied to cover the dural defects, followed by suturing the skin incision (Fig. 5a and Fig. S23). Digital images of the skin wounds and dural defects in the cranial parietal regions at different time points for different groups were recorded (Fig. S24 and Fig. 5b). At 1-week post-operation, the defect was still clearly visible, and significant CSF leakage was observed around the skin incision in the no-treatment group. In the fibrin glue group, a small amount of exudation and residual material could also be seen on the surface of the dural defect. In the 4aPEG-OPA/4aPEG-SSNH_2_ and 4aPEG-OPA/gelatin hydrogel groups, the dural defect areas were covered by the hydrogels, and no CSF exudation was observed in either group. At 2 weeks, the dural defects in the no-treatment group and the fibrin glue group were still visible. In contrast, after treatment with the 4aPEG-OPA/4aPEG-SSNH_2_ or 4aPEG-OPA/gelatin hydrogel, the defect area was covered by fibrous connective tissue. At 3 weeks, the defect areas of all four groups were covered without skin infection. However, obvious congestion was observed in the no-treatment and fibrin glue groups but was absent in the 4aPEG-OPA/4aPEG-SSNH_2_ and 4aPEG-OPA/gelatin groups. These results suggested a local inflammatory response in the no-treatment and fibrin glue groups.

Moreover, magnetic resonance imaging (MRI) was performed on rabbits at 1 week and 2 weeks after the operation to monitor the CSF leakage in each group, as shown in Fig. 5c [71,72]. In this experiment, both CSF and hydrogels showed long T1 (black) and long T2 (white) signals in the images. At 1 week, obvious long T1 and long T2 signals were observed in a large area of the subcutaneous cavity in the no-treatment group and the fibrin glue group, suggesting CSF leakage. In contrast, there was no obvious CSF leakage after treatment with the 4aPEG-OPA/4aPEG-SSNH_2_ and 4aPEG-OPA/gelatin hydrogels, and only long T1 and long T2 signals of the hydrogels were found in the images. At 2 weeks, the areas of long T2 signals were significantly reduced in the no-treatment group and the fibrin glue group, indicating a decrease in CSF leakage. Notably, no obvious swelling was found for the 4aPEG-OPA/gelatin hydrogel after treatment for 2 weeks, which can prevent compression of the brain tissue [31–34].

We analyzed the white blood cell (WBC) count and the contents of glucose, chloride, and total protein in the CSF of rabbits after the operation (Fig. S25). The results showed that the WBC counts for the fibrin glue group and no-treatment group were elevated when compared to those for the normal group, indicating the possibility of infection due to CSF leakage in these two groups. In comparison, the group treated with the 4aPEG-OPA/4aPEG-SSNH_2_ or 4aPEG-OPA/gelatin hydrogel showed no obvious difference in WBC count compared to the normal group. Additionally, there were no significant differences in the contents of glucose, chloride, and total protein in the CSF among the different groups.

The repair of dural defects was analyzed by H&E and Masson's trichrome staining, as shown in Fig. 5d. At 1 week, the dural defect in each group was clearly visible. After treatment with the 4aPEG-OPA/4aPEG-SSNH_2_ or 4aPEG-OPA/gelatin hydrogels, the dural defects were sealed by the hydrogels at 1 week, and the formation of new dural tissue and collagen fiber deposition were found in the dural defect area at 2 weeks. Notably, at 3 weeks, treatment with the 4aPEG-OPA/gelatin hydrogel markedly promoted collagen deposition and the formation of new dura mater similar to normal dural tissue. Nevertheless, no collagen deposition was observed in the area of the dural defect in the no-treatment group at 3 weeks. The deposition of new collagen matrix is the basis for the repair and reconstruction of the dura mater [73]. Thus, these results highlighted the strong potential of the 4aPEG-OPA/gelatin hydrogel for dural defect sealing and repair.

To evaluate the biocompatibility of the hydrogels *in vivo*, routine blood and blood biochemical tests indicated that the hydrogels had no hepatorenal and hematological toxicity at the animal level (Fig. S26). At 1 week, the number of WBCs in blood in the no-treatment group was significantly higher than those in the 4aPEG-OPA/gelatin and 4aPEG-OPA/4aPEG-SSNH_2_ groups, indicating an inflammatory response and a possible infection caused by CSF leakage.

### Sealing lumbar dura mater defects and preventing undesired postoperative adhesion in rabbits

**Figure 6.**
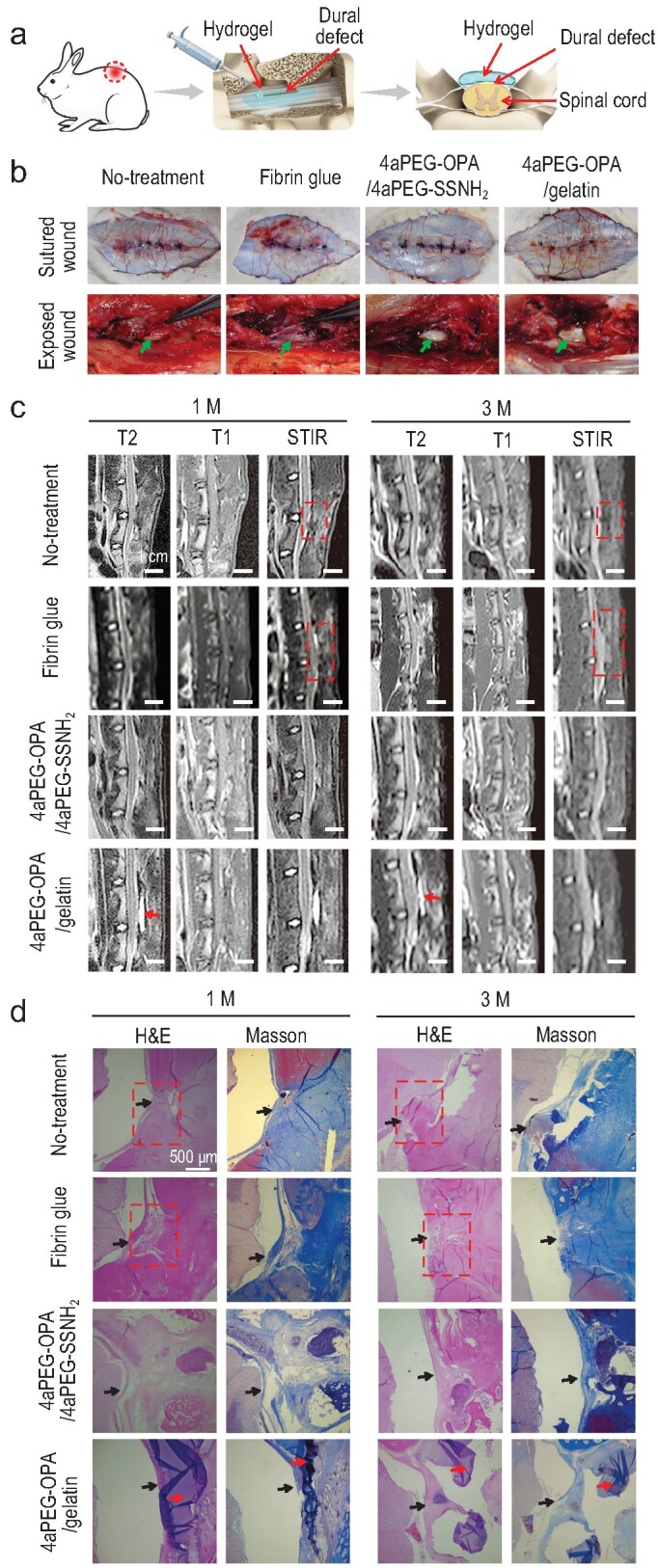
Dural defect sealing and prevention of postoperative adhesion effects of the hydrogel sealants in lumbar dura mater defects of rabbits. (a) Schematic diagram of sealing and preventing undesired postoperative adhesion in the dura mater spinalis of rabbits. (b) Visual inspection of surgical sites to evaluate epidural adhesion at 1 month after different treatments. Green arrows represent the dura mater. (c) The lumbar MRI results of rabbits in the no-treatment, fibrin glue, 4aPEG-OPA/4aPEG-SSNH_2_, and 4aPEG-OPA/gelatin groups. (d) H&E and Masson's trichrome staining for different groups. Yellow arrow: dura mater; red arrow: residual hydrogels; red dashed box: area of epidural adhesion; T1: T1 weighted image; T2: T2 weighted image; STIR: short time of inversion recovery.

The operative treatment of spinal dural tears or CSF leakage often involves laminectomy. Epidural fibrosis and undesired adhesion to epidural tissues are likely to occur after this operation, which cause a series of problems for patients, such as chronic persistent low back pain, radicular pain, and the risk of secondary surgery [74]. In clinical practice, physical isolation is the most commonly used method to solve this problem [75,76]. The hydrogels obtained in this work can act as a physical barrier after complete gelation, which will be beneficial for preventing the postoperative adhesion of dura mater to epidural tissues. Therefore, we further evaluated the performance of the hydrogels for sealing the dural defect and preventing postoperative adhesion in the dura mater spinalis of rabbits (Fig. 6a and Fig. S27). At 1-month post-operation, the dura mater could not be separated normally from the epidural tissues in the no-treatment and fibrin glue groups (Fig. 6b), suggesting serious tissue adhesion. In contrast, after treatment with the 4aPEG-OPA/gelatin hydrogel, the surface of the dura mater was covered with the residual hydrogel at 1 month (Fig. S28). The dura mater was clearly visible after removing the hydrogels, and could be easily separated from the epidural soft tissues, suggesting that the hydrogel could effectively prevent postoperative adhesion during the repair of dural defects.

The prevention of postoperative adhesion by hydrogel treatment was observed by rabbit lumbar MRI at different time points (Fig. 6c). In the no-treatment or fibrin glue group, long T1 and short T2 signals of mass tissue could be observed in the operation site at 1 month, suggesting the formation of scar tissue. The scar tissue was closely adhered to the dura and spinal cord, and the compression of the spinal cord by the scar tissue tended to increase at 3 months. In the 4aPEG-OPA/4aPEG-SSNH_2_ group, the demarcation between the spinal cord and the posterior soft tissues was clear, indicating no obvious adhesion. A small amount of residual hydrogel was observed at 1 month, and the hydrogel was completely degraded at 3 months. For the group treated with 4aPEG-OPA/gelatin hydrogel, the hydrogel remained between the spinal cord and the posterior soft tissues at 1 month, the structure of each layer was quite clear, and no adhesion was observed between the layers. At 3 months, the residual size of the hydrogel was noticeably reduced compared to that at 1 month. The MRI results indicated that the 4aPEG-OPA/gelatin hydrogel could retain its integrity in the presence of body fluid and enzymes for 3 months to act as a physical barrier for preventing postoperative tissue adhesion.

Histological analysis was further conducted by H&E and Masson's trichrome staining. As shown in Fig. 6d, at 1- and 3-months post-operation, extensive scar tissue formation and adhesion were seen in the laminar defects of the no-treatment and fibrin glue groups. In contrast, the groups treated with 4aPEG-OPA/4aPEG-SSNH_2_ and 4aPEG-OPA/gelatin hydrogels showed markedly reduced adhesion between the epidural tissue and the dura. In addition, routine blood and blood biochemical results suggested that the hydrogels exhibited no detectable chronic hepatorenal toxicity or hematological toxicity *in vivo* at 3 months (Fig. S29). This confirmed the good long-term biocompatibility of the 4aPEG-OPA/4aPEG-SSNH_2_ and 4aPEG-OPA/gelatin hydrogels *in vivo*.

## CONCLUSION

In this study, an injectable, low-swelling hydrogel sealant was developed via catalyst-free OPA/amine chemistry for dural sealing and repair as well as preventing postoperative adhesion. The OPA end groups of 4aPEG-OPA could react with the amine groups of gelatin to form a cross-linking network, and also couple with the amine groups on the dural surface for sealing the defect. The 4aPEG-OPA/gelatin hydrogel showed appropriate gelation time, good mechanical properties, biodegradability, and biocompatibility. Especially, the 4aPEG-OPA/gelatin hydrogel exhibited a low swelling ratio under physiological conditions for avoiding compression of the spinal cord. The hydrogel showed an over 3-fold higher adhesive strength than a commercially-available fibrin glue. After administration to the lumbar and cerebral dural defects of rat and rabbit models, the 4aPEG-OPA/gelatin hydrogels effectively sealed the dural defects, achieving watertight closure of the wounds and preventing CSF leakage, without detectable compression on the central nervous system. Moreover, the treatment with hydrogels reduced local inflammation, epidural fibrosis, and postoperative adhesion of dural defect areas. Therefore, these results demonstrated the strong potential of the 4aPEG-OPA/gelatin hydrogel as a multifunctional sealant for efficient sealing of dural defects and prevention of postoperative adhesion.

## ETHICAL STATEMENTS

The animal experiments in this study were carried out according to the laboratory animal welfare Chinese National Standard (GB/T 35892–2018) and approved by the Animal Ethics Committee of College of Basic Medicine Sciences, Jilin University (2022–110) and Changchun Institute of Applied Chemistry, CAS (2021–54).

## Supplementary Material

nwae160_Supplemental_File
