# Supplementary material for: A low-swelling hydrogel as a multirole sealant for efficient dural defect sealing and prevention of postoperative adhesion
Source: Natl Sci Rev. 2024 Apr 30;11(6):nwae160. doi: 10.1093/nsr/nwae160 (PMC11168225; doi:10.1093/nsr/nwae160)
Supplement: nwae160_Supplemental_File [file nwae160_supplemental_file.pdf]

Supplementary Data for:

A low-swelling hydrogel as a multirole sealant for efficient dural defect sealing and prevention of postoperative adhesion

Xueliang Cheng[1,3,†], Zhen Zhang[1,†], Hui Ren[1,2], Zheng Zou[1,2], Yu Zhang[1], Yang Qu[3], Xuesi Chen[1,2,*], Jianwu Zhao[3,*] and Chaoliang He[1,2,*]

[1]CAS Key Laboratory of Polymer Ecomaterials, Changchun Institute of Applied Chemistry, Chinese Academy of Sciences, Changchun 130022, China;

[2]School of Applied Chemistry and Engineering, University of Science and Technology of China, Hefei 230026, China;

[3]Department of Orthopedics, The Second Norman Bethune Hospital of Jilin University, Changchun 130014, China

***Corresponding authors**. E-mails: xschen@ciac.ac.cn; jianwu@jlu.edu.cn; clhe@ciac.ac.cn

†Equally contributed to this work.

Experimental Section

1. Materials

Gelatin was purchased from Sigma–Aldrich (Missouri, United States). Four-armed PEG (M_n = 10 kDa) was purchased from Pharmicell Co., Inc. (Seoul, Republic of Korea). Cystamine dihydrochloride was purchased from TCI (Tokyo, Japan). Fibrin glue (Bioseal) was obtained from Bioseal Biotech Ltd. (Guangzhou, China), an affiliated enterprise of Johnson & Johnson (United States). Dulbecco's modified Eagle medium (DMEM, MA0212) and Cell Counting Kit-8 (CCK-8, MA0218) were obtained from Meilunbio (Dalian, China). The Calcein/PI Cell Viability/Cytotoxicity Assay Kit (C2015M) was purchased from Beyotime Biotechnology (Shanghai, China). Trypsin (T1300) and penicillin/streptomycin (P1400) were purchased from Solabio (Beijing, China). Newborn calf serum (80230-641) was purchased from Sijiqing (Huzhou, China). Sterile PBS was purchased from Servicebio (Wuhan, China). Pentobarbital sodium was purchased from Dingguo Biotechnology (Changchun, China). ANTAI anesthetic was purchased from DMK Biological Technology (Jilin, China). Japanese white rabbits were purchased from Changchun Yisi Experimental Animal Technology (Changchun, China), and Sprague−Dawley rats were purchased from Liaoning Changsheng Biotechnology (Benxi, China).

2. Synthesis of 4aPEG-OPA and 4aPEG-SSNH$_2$

4aPEG-OPA was synthesized according to our previously reported method [1]. 4aPEG-SS-NH$_2$ was synthesized as follows. First, 4-armed PEG (5.0 g, 0.5 mmol) was dissolved in dry dichloromethane (30 mL), and then NPC (2.0 g, 10 mmol) in

dichloromethane (20 mL) was slowly added. The mixture was stirred at 25 ℃ for 12 h. After the reaction was completed, it was washed three times with saturated brine, and the organic phase was dried with anhydrous sodium sulfate. After filtration and concentration, the solution was poured into ice-cold diethyl ether to obtain a white powder (4.5 g, 90% yield). Then, the powder (2.0 g, 0.2 mmol) was dissolved in dichloromethane (30 mL) and added dropwise to a solution of cystamine (1.2 g, 8.0 mmol) in dichloromethane (20 mL) in an ice bath. The mixture was stirred at 25 ℃ for 12 h. After that, the mixture was washed with brine and dried with anhydrous sodium sulfate. The solution was filtered, concentrated and precipitated to obtain a light-yellow powder (1.7 g, yield 85%).

3. Preparation of the hydrogels

A 10% (w/v) gelatin solution in PBS (pH 7.4) was incubated in a 37 ℃ water bath before use. The 4aPEG-OPA/gelatin hydrogels were prepared by mixing 10% (w/v) 4aPEG-OPA with 10% (w/v) gelatin at a volume ratio of 1:1. The 4aPEG-OPA/4aPEG-SSNH$_2$ hydrogels were prepared by mixing 10% (w/v) 4aPEG-OPA with 10% (w/v) 4aPEG-SSNH$_2$ with an OPA/NH$_2$ molar ratio of 1:1. For cell and animal experiments, the precursor solutions were sterilized by filtration through 0.2 μm filters.

4. Gelation time

The gelation time of the hydrogel was tested by the inversion method at room temperature (25 ℃) and 37 ℃. The 10% (w/v) precursor solutions in PBS were added to a vial at a volume ratio of 1:1 and fully mixed. The gelation time was determined as

the time when no flow was observed after inverting the vial.

5. Rheological tests

The rheological properties of the hydrogel were measured by a rheometer (MCR 301, Anton Paar GmbH) equipped with a parallel plate of 50 mm diameter at a gap of 0.5 mm. The precursor solutions in PBS were mixed and then pipetted onto the bottom plate of the rheometer at 37 ℃. The change in the storage modulus and loss modulus of the sample with time was recorded at a fixed frequency of 1 Hz and strain of 1%. The edge of the gap was sealed by a thin layer of silicon oil to prevent water evaporation.

6. Cryo-SEM imaging

The micromorphology of the hydrogel was observed by cryo-SEM (Sigma 300, ZEISS). The sample was cryo-fixed in slush nitrogen (-210 ℃) for approximately 30 seconds. After that, the sample was transferred into a cryopreparation chamber and fractured to create a fresh surface for observation. Then, the sample was sublimated at -90 ℃ for 10 min and sprayed with gold at -140 ℃. Finally, the sample was transferred into a microscope chamber and photographed. The working voltage was 5 kV.

7. Compression tests

Cylindrical samples with an inner diameter of 8 mm and a height of 5 mm were prepared in custom-made PTFE molds and incubated at 37 ℃ for 2 h for gelation. The compression test was carried out by using a universal testing machine (AGS-X 50N, SHIMADZU) at a rate of 2 mm/min at room temperature. The compressive modulus

was calculated as the slope of the stress-strain curve between a strain of 10% and 20%. Five replicate tests were performed for each material. To further evaluate the fatigue resistance of the hydrogels, cyclic compression tests were performed under the same conditions. The number of cycles was ten.

8. Tensile tests

Cuboid samples with a width of 5 mm, thickness of 2 mm, and length of 30 mm were prepared in custom-made silicon molds. The tensile tests were carried out by using a universal testing machine (AGS-X 50N, SHIMADZU) at a rate of 2 mm/min at room temperature. The tensile modulus was calculated as the slope of the stress-strain curve between a strain of 10% and 20%. Seven replicate tests were performed for each material.

9. *In vitro* adhesive strength

First, porcine casing was fixed on a glass slide with an industrial adhesive (ethyl cyanoacrylate (502), Tonglin Adhesives, Harbin, China). The hydrogel precursor solutions were mixed and spread immediately to the overlapping area of the porcine casing (2.5 cm × 1 cm). After that, a weight of 200 g was placed on the overlapping area for 0.5 h at 37 ℃ under humid conditions. The adhesive strength was determined by a universal testing machine at a speed of 2 mm/min. Six replicate tests were performed for each material.

10. *In vitro* burst pressure tests

To evaluate the burst pressure of the hydrogel sealants, a hole 1.6 mm in diameter was made in a porcine casing by a needle. After that, 50 μL of the hydrogel precursors

were mixed and added immediately onto the porcine casing. Notably, before adding materials, it was necessary to ensure that the liquid level of PBS consistently reached the hole and that the surface of the porcine casing was slightly wet with PBS to simulate actual clinical scenarios. The samples were incubated for 5 min, 15 min and 30 min at 37 ℃ under wet conditions to form hydrogels approximately 1 mm thick. Then, PBS was pumped continuously and steadily at a rate of 0.8 mL/min by a syringe pump, and the burst pressure was recorded by a pressure gauge. Six replicate tests were performed for each material.

11. *In vitro* swelling and degradation of the hydrogels

A 100 μL hydrogel was prepared in a 2 mL centrifuge tube, and 1 mL degradation medium was added. To evaluate the enzymatic degradation of the 4aPEG-OPA/gelatin hydrogel, PBS containing collagenase (5 U/mL or 10 U/mL) was used as the degradation medium. The tube was placed in an oscillating incubator at 37 ℃. At different time points, the degradation medium was removed, and the weights of the hydrogels were obtained. The swelling ratio was calculated according to the following formula: swelling ratio (%) = $(W_t - W_0)/W_0 \times 100\%$ [2, 3]. W_0 was the initial weight of the hydrogel, and W_t was the residual weight at different time points.

12. Biocompatibility of the hydrogels

12.1 *In vitro* cytotoxicity tests

First, we obtained the extracts of the hydrogels according to the national medical device standard ISO 10993-5 [4]. The prepared hydrogels were soaked in DMEM at 200 mg/mL and placed in an oscillating incubator at 37 ℃ for 24 h to obtain the

hydrogel extracts. NIH 3T3 (ATCC) cells in the logarithmic growth phase were seeded in 96-well plates (5000 cells per well). After incubation for 24 h, the culture medium was replaced with twofold serial dilutions of the hydrogel extracts (100%, 50%, 25%, 12.5%, 6.25%), and the cell viability was detected at different time points (24 h, 48 h, 72 h) by CCK-8 assay according to the manufacturer's protocol. The cytotoxicity of the hydrogels was also evaluated by live-dead staining. NIH 3T3 cells were inoculated in 24-well plates (15000 cells per well). After incubation with the hydrogel extracts for 72 h, the cells were stained and photographed according to the instructions of the manufacturer of the live-dead staining kit.

12.2 *In vitro* hemolysis tests

The prepared hydrogels were soaked in 0.9% normal saline at 200 mg/mL and placed in an oscillating incubator at 37 ℃ for 24 h to obtain the hydrogel extracts. After collecting rabbit venous blood, red blood cells were obtained by repeated centrifugation and washing, and then normal saline was added to obtain a 2% cell suspension. Then, the red blood cell suspension was mixed with the twofold serial dilution of the hydrogel extracts at a volume ratio of 1:1. The negative control was formed by the addition of 0.9% normal saline, and the positive control was formed by the addition of pure water. The mixed solutions were incubated for 1 h and 3 h. Hemolysis was recorded by a digital camera, and the absorbance value at 540 nm was measured by a microplate reader (Spark, Tecan) to calculate the hemolysis ratio. In addition, the morphology of red blood cells was observed by optical microscopy.

13. Animal procedures

The animal experimental schemes and care in this study were carried out according to the laboratory animal welfare Chinese National Standard (GB/T 35892-2018) and approved by the Animal Ethics Committee of College of Basic Medicine Sciences, Jilin University (2022-110) and Changchun Institute of Applied Chemistry, CAS (2021-54). Male Sprague Dawley rats (250 g) and male Japanese white rabbits (2.5 kg) were purchased from Yisi Experimental Animals Technology Limited (Changchun, China). The animals were housed individually under the following conditions: temperature, 23-25 ℃; relative humidity, 60.0-70.0%; and interval of illumination, 12 h. All animals were adapted to the conditions of the animal room for a week prior to initiating any experiments.

14. *In vivo* degradation and histocompatibility of the hydrogels

The *in vivo* degradation and histocompatibility of the hydrogels was evaluated by subcutaneous implantation experiments. Rats were anesthetized by the intraperitoneal injection of pentobarbital sodium and shaved. A skin incision approximately 1 cm in length was made on each side of the back of the rat, and a subcutaneous pocket was created with blunt forceps for implantation of the hydrogels. After that, a preprepared 150 μL sterile cylindrical hydrogel was implanted into each pocket. The wound was sutured and sterilized. At the scheduled time point, rats were euthanized. Local inflammatory reactions were observed, and the hydrogels together with the surrounding tissues were excised for histological analysis. The remaining hydrogels were weighed for degradation curves (three samples for each time point).

15. Dural defect sealing performance of the hydrogels

After anesthesia with ANTAI anesthetic, a longitudinal incision was made along the sagittal suture of the rabbits to fully expose the skull. A region of the skull 10 mm in diameter was removed with a TRAUS 204 electric grinder, and a dural defect 5 mm in length was created with microscissors. The dural defect model was established by confirming CSF leakage from the defect. Then, the indwelling needle was embedded in the subarachnoid space at the top of the forehead bone defect. The liquid outflow speed of the dural defects on both sides was controlled by adjusting the position and direction of the indwelling needle to ensure that the liquid outflow rate on each side was similar. After that, hydrogels or fibrin glue (100 μL) was added on each side of the sagittal suture to seal the dural defect. After 5 minutes, PBS was infused into the subarachnoid space through the indwelling needle at a speed of 0.8 mL/min to evaluate the sealing effect of different sealants.

In addition, considering that CSF leakage is continuous in the real scenario, we investigated the instant sealing performance of the hydrogels. In this experiment, PBS was continuously injected at a speed of 0.8 mL/min, and the pressure was maintained at approximately 10 cmH_2O to simulate CSF circulation. When the defect was actively leaking, precursor solutions of hydrogels or fibrin glue (100 μL) were added to evaluate whether the hydrogels could effectively seal the dural defect with continuous liquid exudation.

16. Sealing and repair of lumbar dura mater defects in a rat model

The rats (approximately 250 g) were used to assess the sealing and repair of lumbar dural defects with the hydrogel sealants. After anesthesia with pentobarbital sodium,

the backs of the rats were shaved and disinfected. The skin, subcutaneous tissues, and muscle were incised along the mid-lumbar segment of the back. The spinous process and lamina were removed with single-joint bone scissors, and the dural sac was exposed. The dural sac pulsation was visible under direct vision. A dural defect approximately 1 cm in length was created by longitudinally cutting the dura mater and arachnoid membrane with micro hook forceps and micro scissors. The successful establishment of a dural defect model was confirmed when clear CSF flowed out. The rats were randomly divided into four groups: no-treatment group, fibrin glue group, 4aPEG-OPA/4aPEG-SSNH$_2$ group, and 4aPEG-OPA/gelatin group (six animals per group). Hydrogel or fibrin glue (60 μL) was added to seal the dural defects, after which the muscle and skin were sutured layer by layer. The no-treatment group was sutured directly following creation of the dural defects. Rats with neurological dysfunction immediately after the operation were euthanized. All animals could eat, drink and urinate normally after the operation. The rats were euthanized at 1 week and 2 weeks after the operation. The tissues were collected for histological analysis. The expression levels of inflammatory factors (IL-6, TNF-α) in the spinal cord and surrounding tissues were determined by ELISA kits.

17. Sealing and repair of cerebral dura mater defects in a rabbit model

Male Japanese white rabbits (2.5 kg) were used to assess the sealing and repair of cerebral dural defects with hydrogel sealants. After anesthesia with ANTAI anesthetic, the skin on the top of the rabbit's forehead was shaved and disinfected. A longitudinal incision of approximately 3 cm length was made to expose the skull. A region of the

skull 10 mm in diameter was removed with a TRAUS 204 electric grinder, and a dural defect 5 mm in length was created with microscissors. The rabbits were randomly divided into four groups: no-treatment group, fibrin glue group, 4aPEG-OPA/4aPEG-$SSNH_2$ group, and 4aPEG-OPA/gelatin group (four animals per group). Hydrogels or fibrin glue (100 μL) was added to form sealants of 1-1.5 mm in thickness to seal the dural defects. Then, the skin incision was sutured and disinfected. Rabbits were given an intramuscular injection of ceftriaxone sodium every day for 4 days to prevent infection. Magnetic resonance imaging (MRI, UMR 560, 1.5 T) was used to monitor CSF leakage at 1 week and 2 weeks postoperation [5]. Venous blood was collected from the rabbits for blood biochemical analysis. Finally, the rabbits were euthanized. The tissues at the surgical sites were processed for histological assessment, and the CSF was collected for biochemical analysis [6-8].

18. Sealing and anti-postoperative adhesion effect of the hydrogel sealants in lumbar dura mater defects in a rabbit model

Male Japanese white rabbits (2.5 kg) were used. After anesthesia with ANTAI anesthetic, the L4-L5 spinous process was located. The skin and subcutaneous tissues over the L4-L5 spinous process were incised, and bilateral perispinous muscles were separated to fully expose the spinous process and lamina. After removing the lamina and exposing the dural sac, a defect approximately 10 mm in length was created by cutting the dura mater and arachnoid membrane with microscissors. The rabbits were randomly divided into four groups: no-treatment group, fibrin glue group, 4aPEG-OPA/4aPEG-$SSNH_2$ group, and 4aPEG-OPA/gelatin group (four animals per

group). Hydrogel or fibrin glue (150 μL) was added to form sealants of 1-1.5 mm in thickness to seal the dural defects. The muscle and the skin were then sutured layer by layer. At 1 month and 3 months after the operation, MRI (UMR 560, 1.5 T) was used to monitor the CSF leakage, and the venous blood of rabbits was collected for blood biochemical analysis. Finally, the rabbits were euthanized, and the tissues were collected for histological analysis.

19. Statistical analysis

All the data are expressed as the mean ± standard deviation (SD). All statistical analyses were performed using SPSS 25.0. The statistical significance of the difference between two groups was assessed by *t* test, and multiple groups were analyzed using one-way analysis of variance with Tukey's post hoc test. Values of $P < 0.05$ were considered statistically significant.

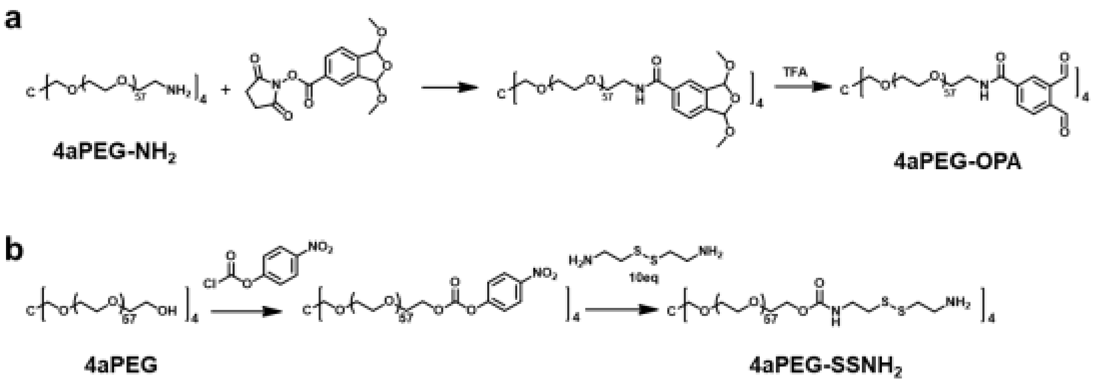

Figure S1. Synthesis routes for (a) OPA-terminated 4aPEG (4aPEG-OPA) and (b) cystamine-modified 4aPEG (4aPEG-SSNH$_2$).

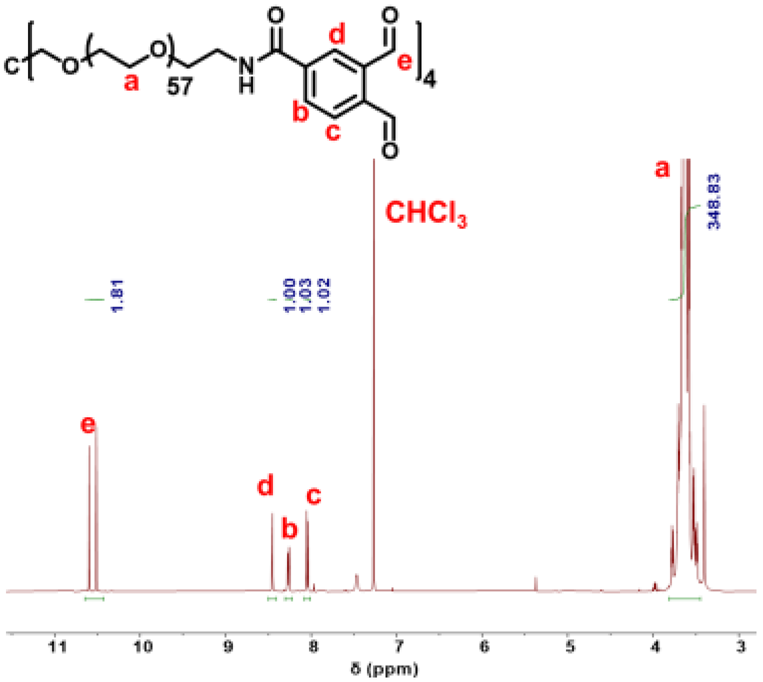

Figure S2. ^{1}H NMR spectrum of 4aPEG-OPA in $CDCl_3$.

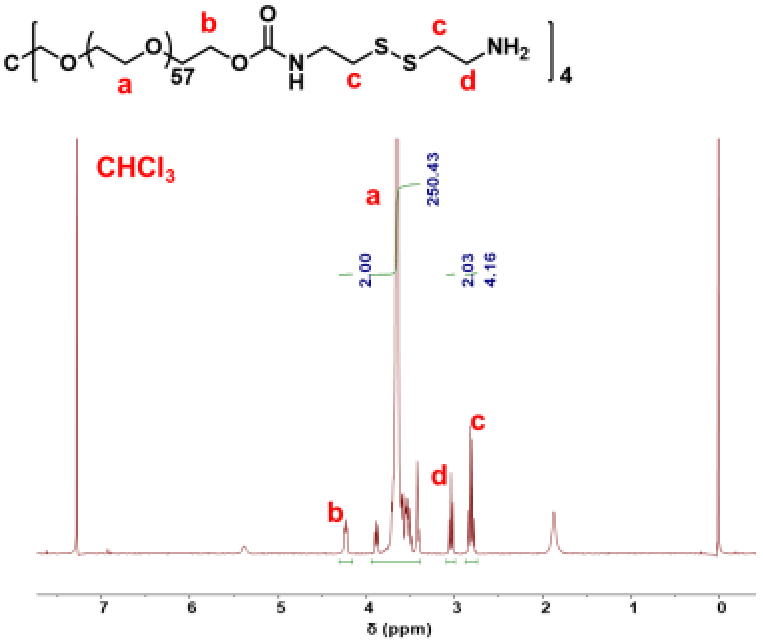

Figure S3. ^{1}H NMR spectrum of 4aPEG-$SSNH_2$ in $CDCl_3$.

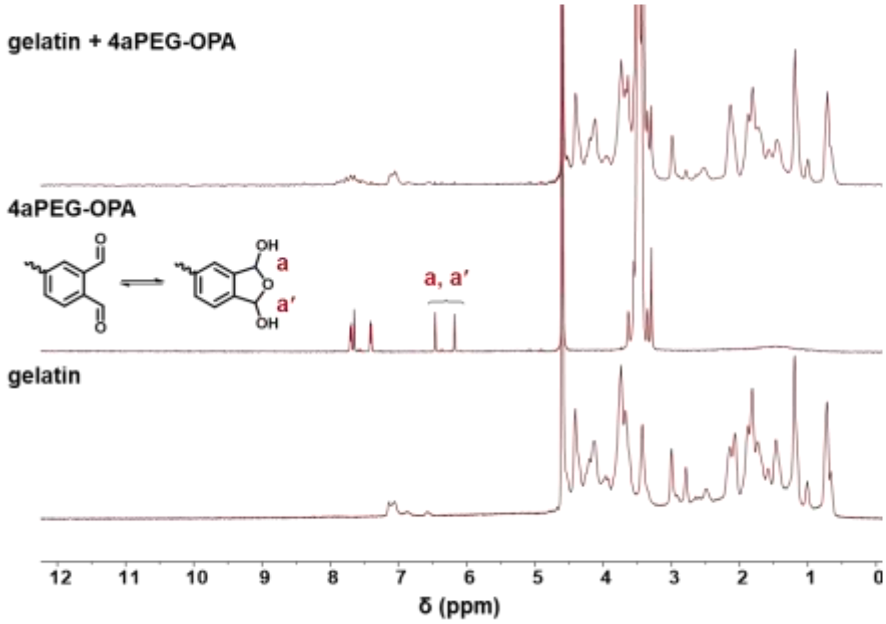

Figure S4. ^{1}H NMR spectrum of gelatin, 4aPEG-OPA and the mixture of gelatin and 4aPEG-OPA for 24 h in D_2O.

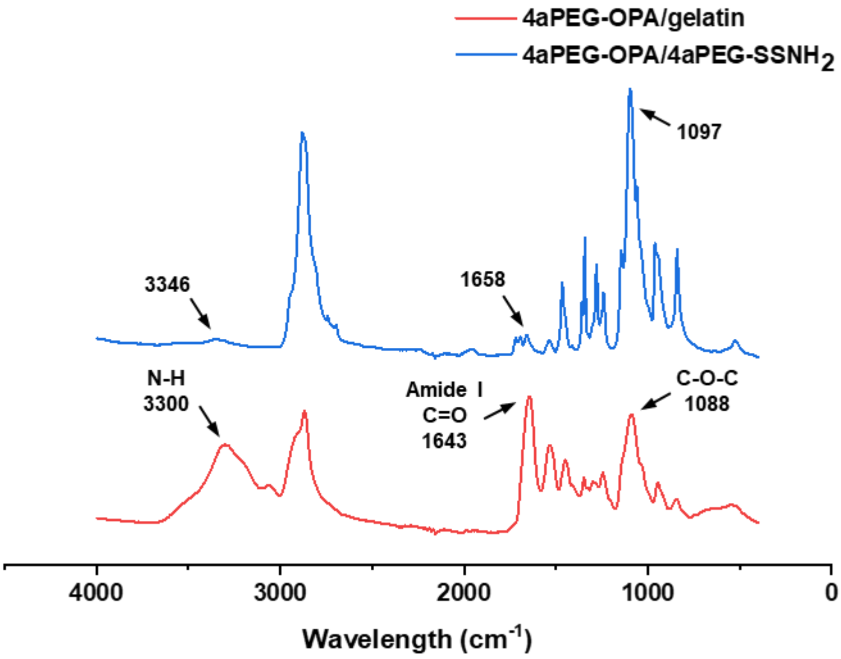

Figure S5. FTIR spectrum of the 4aPEG-OPA/gelatin and 4aPEG-OPA/4aPEG-$SSNH_2$ hydrogels.

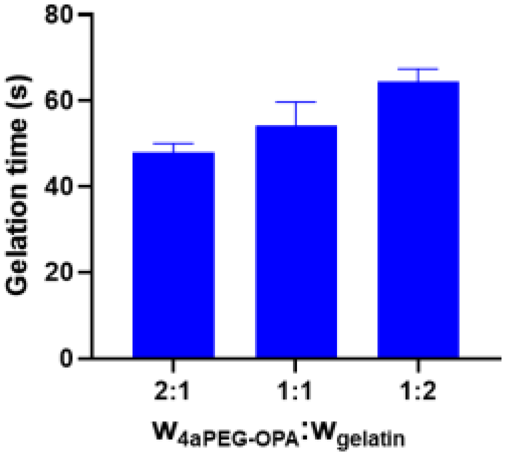

Figure S6. Gelation time of 4aPEG-OPA/gelatin hydrogel with 4aPEG-OPA:gelatin weight ratios of 2:1, 1:1, and 1:2 at 37 ℃.

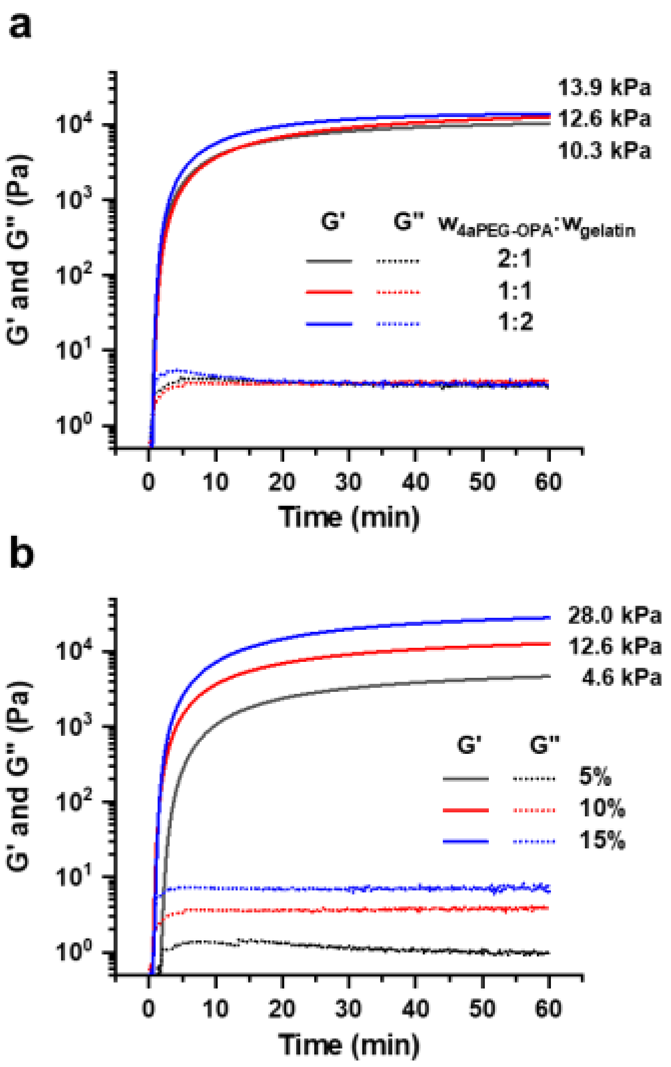

Figure S7. (a) Time-sweep rheological tests of the 10% (w/v) mixture of 4aPEG-OPA and gelatin with 4aPEG-OPA:gelatin weight ratios of 2:1, 1:1, and 1:2. (b) Time-sweep rheological tests of the 1:1 (w/w) mixture of 4aPEG-OPA and gelatin with polymer concentrations of 5%, 10%, and 15% (w/v).

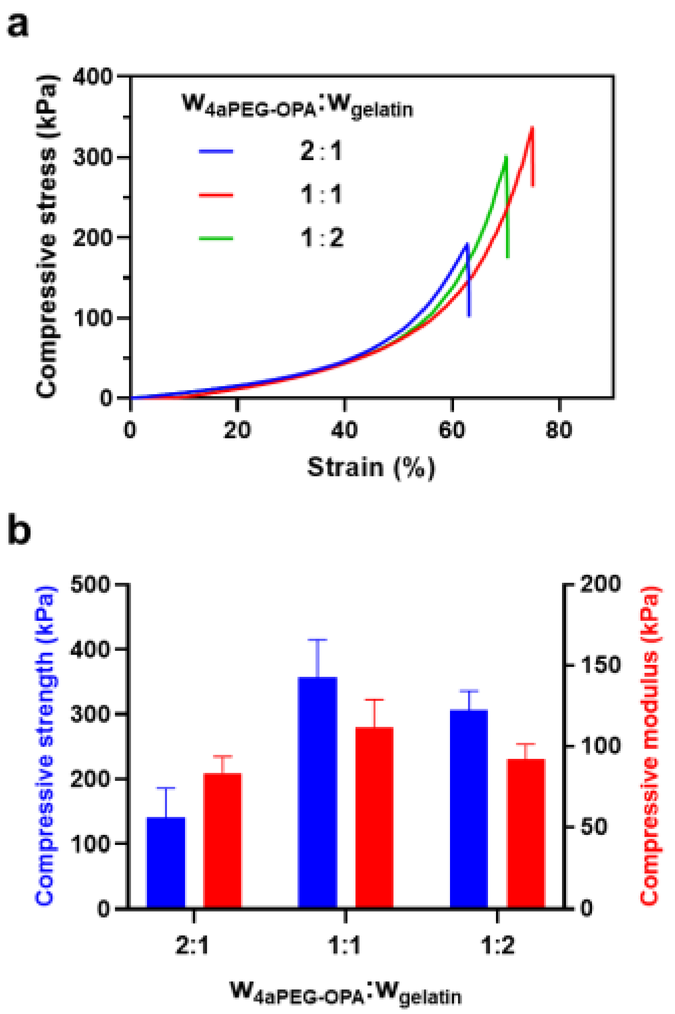

Figure S8. **(**a) Representative compressive stress–strain curves of 4aPEG-OPA/gelatin hydrogel with 4aPEG-OPA:gelatin weight ratios of 2:1, 1:1, and 1:2. (b) Compressive strengths and moduli.

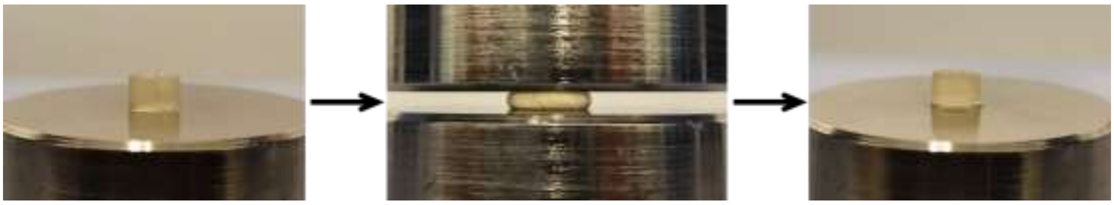

Figure S9. The 4aPEG-OPA/gelatin hydrogel maintained the structural integrity after compression test.

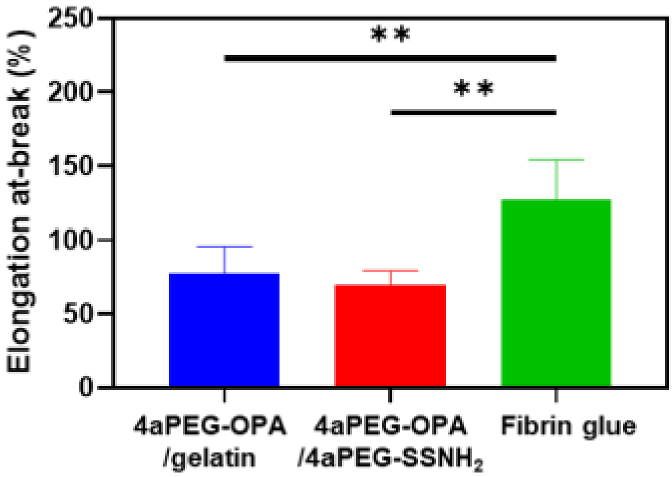

Figure S10. The elongation at-break of 4aPEG-OPA/gelatin, 4aPEG-OPA/4aPEG-$SSNH_2$, fibrin glue. ($n = 7$). **$P < 0.01$.

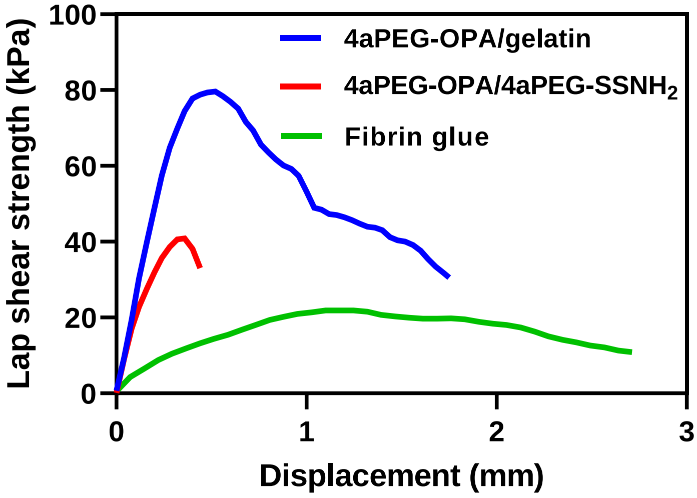

Figure S11. The lap shear curves of 4aPEG-OPA/gelatin, 4aPEG-OPA/4aPEG-SSNH$_2$, fibrin glue.

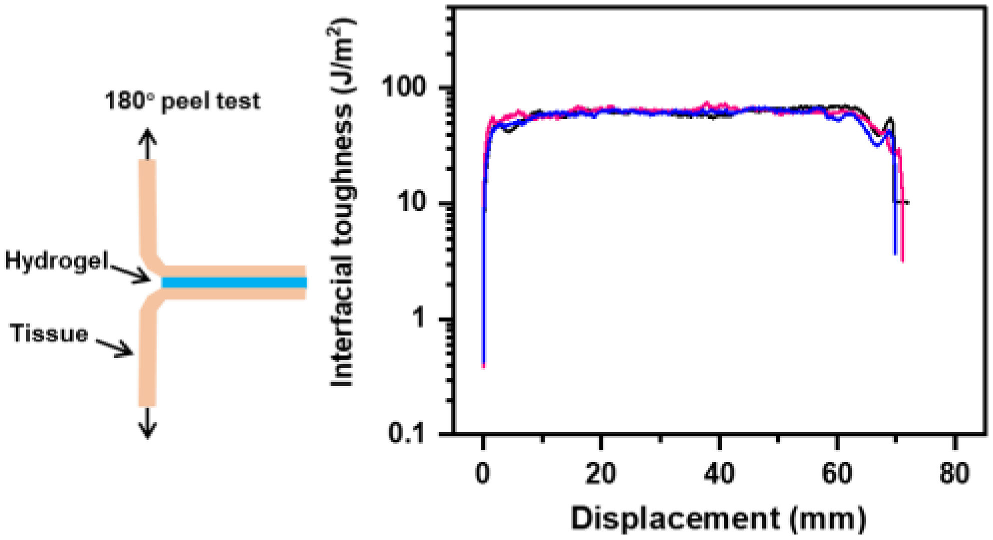

Figure S12. The interfacial toughness of 4aPEG-OPA/gelatin hydrogel determined by 180 °peel test on porcine skin (ASTM F2256).

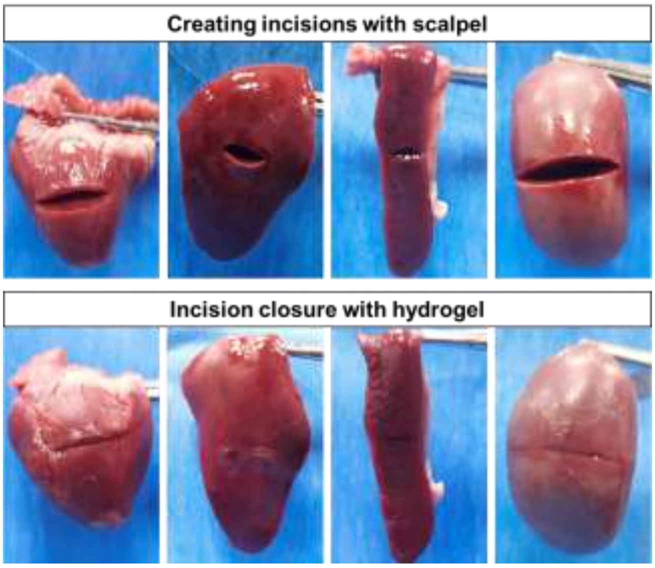

Figure S13. Photographs showing the tissue adhesion performance of 4aPEG-OPA/gelatin hydrogel on the heart, liver, spleen, and kidney of rabbit.

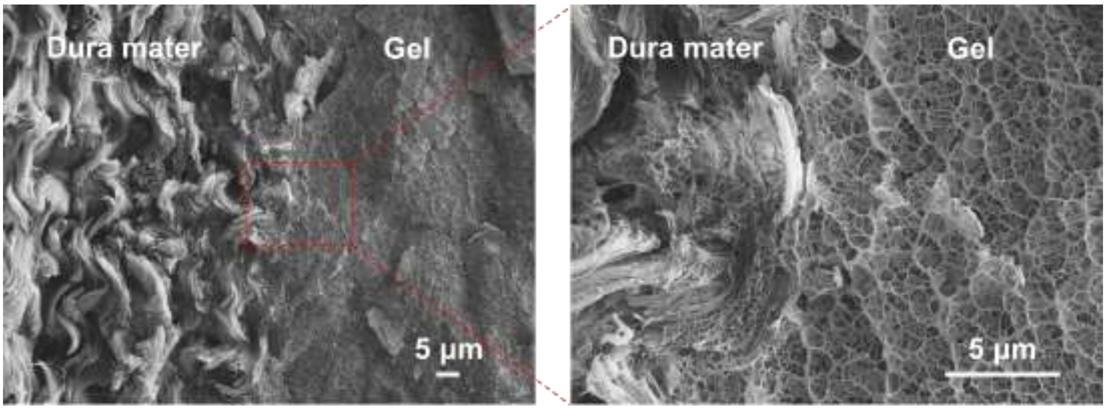

Figure S14. Cryo-SEM images of the interface between 4aPEG-OPA/gelatin hydrogel and porcine cerebral dura mater.

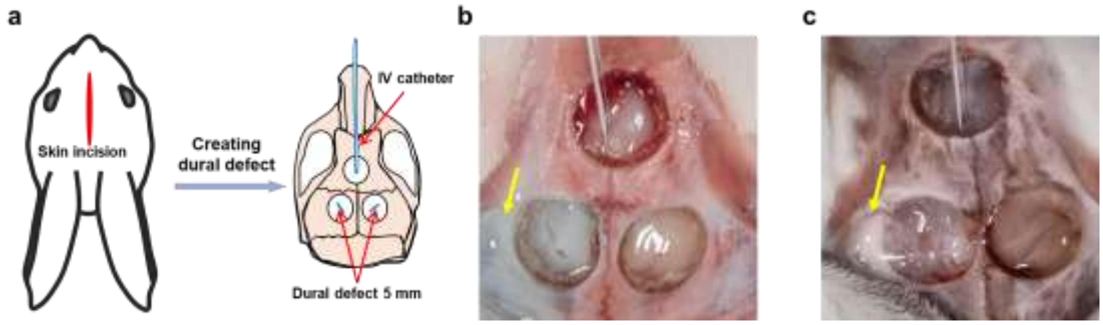

Figure S15. (a) Schematic illustration of the rabbit model of cerebral dural defects. PBS was pumped into the subarachnoid space through an indwelling needle after adding the sealants for 5 minutes. (b) Sealing effect of fibrin glue (left) and 4aPEG-OPA/gelatin (right) on dural defects in rabbits (yellow arrow: leaking PBS). (c) Sealing effect of fibrin glue (left) and 4aPEG-OPA/4aPEG-SSNH$_2$ (right) on dural defects in rabbits (yellow arrow: leaking PBS).

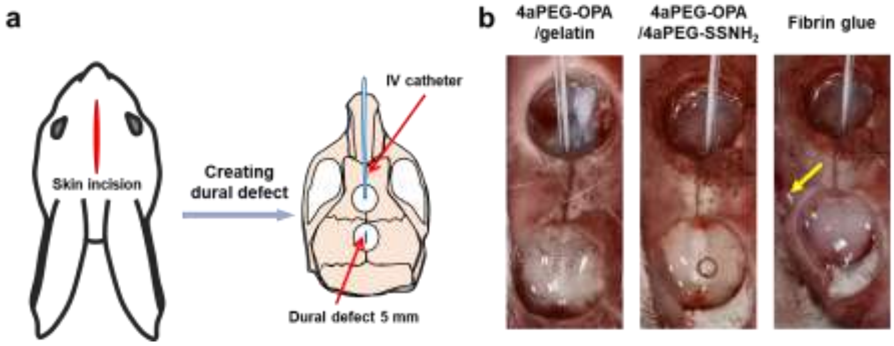

Figure S16. (a) Schematic illustration and (b) images of instant sealing effect of 4aPEG-OPA/gelatin, 4aPEG-OPA/4aPEG-SSNH$_2$ or fibrin glue on dural defect in rabbits. The sealants were added in the case of persistent PBS pumping at the dural defect (yellow arrow: leaking PBS).

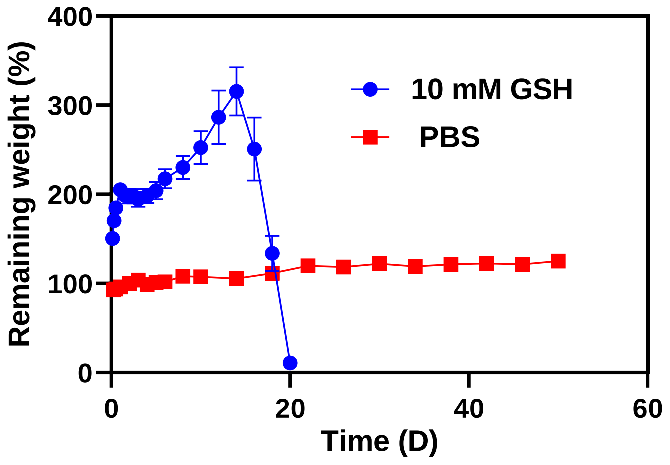

Figure S17. Degradation of 4aPEG-OPA/4aPEG-SSNH$_2$ hydrogel in PBS and 10 mM GSH.

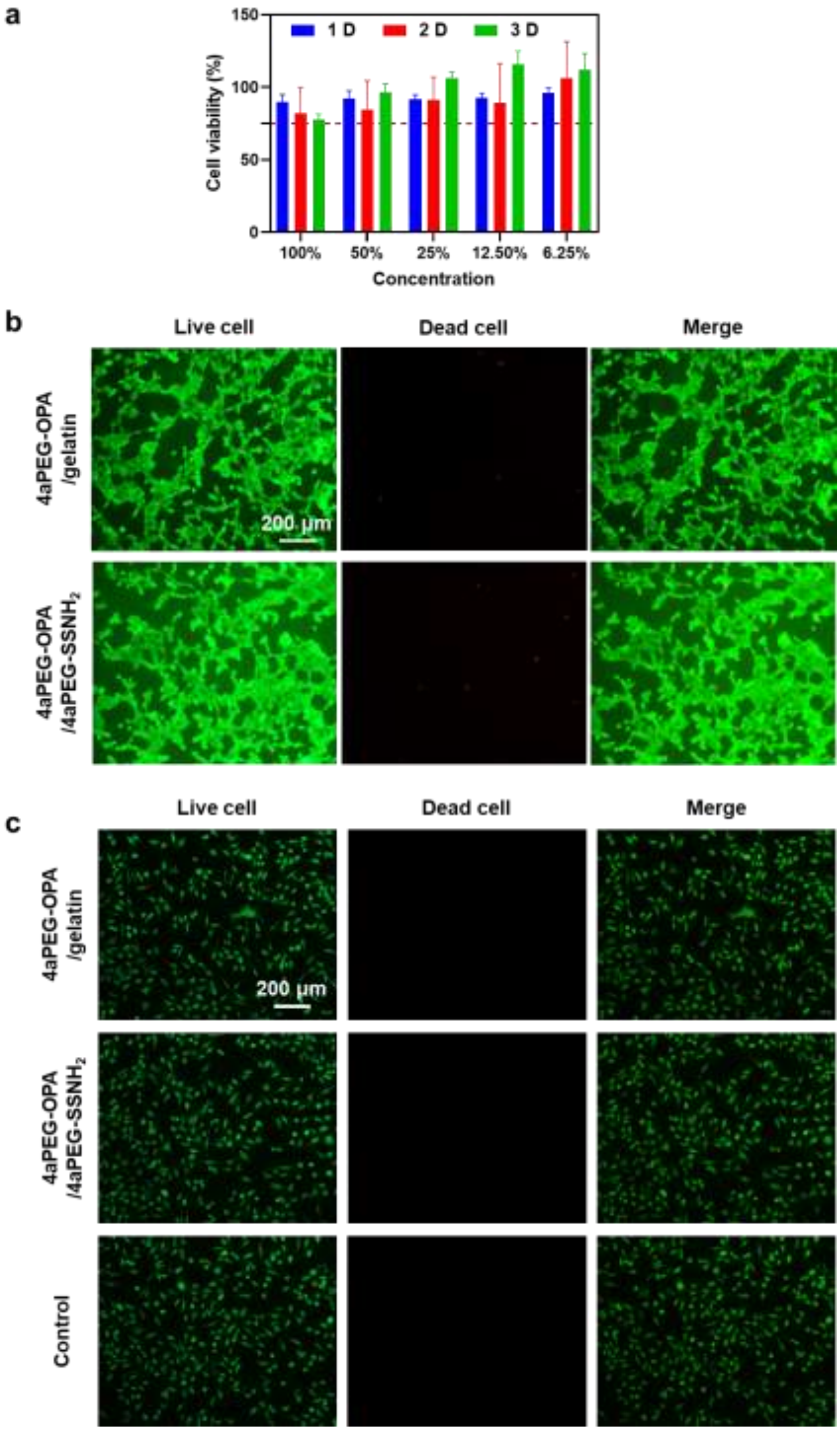

Figure S18. (a) Cell viability of NIH 3T3 cells incubated with 4aPEG-OPA/4aPEG-SSNH$_2$ extract for 1 D, 2 D, and 3 D. (b) Live and dead staining of NIH 3T3 cells incubated with 4aPEG-OPA/gelatin and 4aPEG-OPA/4aPEG-SSNH$_2$ extract for 3 D. (c) Live and dead staining of L929 cells incubated with hydrogel extracts or blank cell culture medium for 3 D.

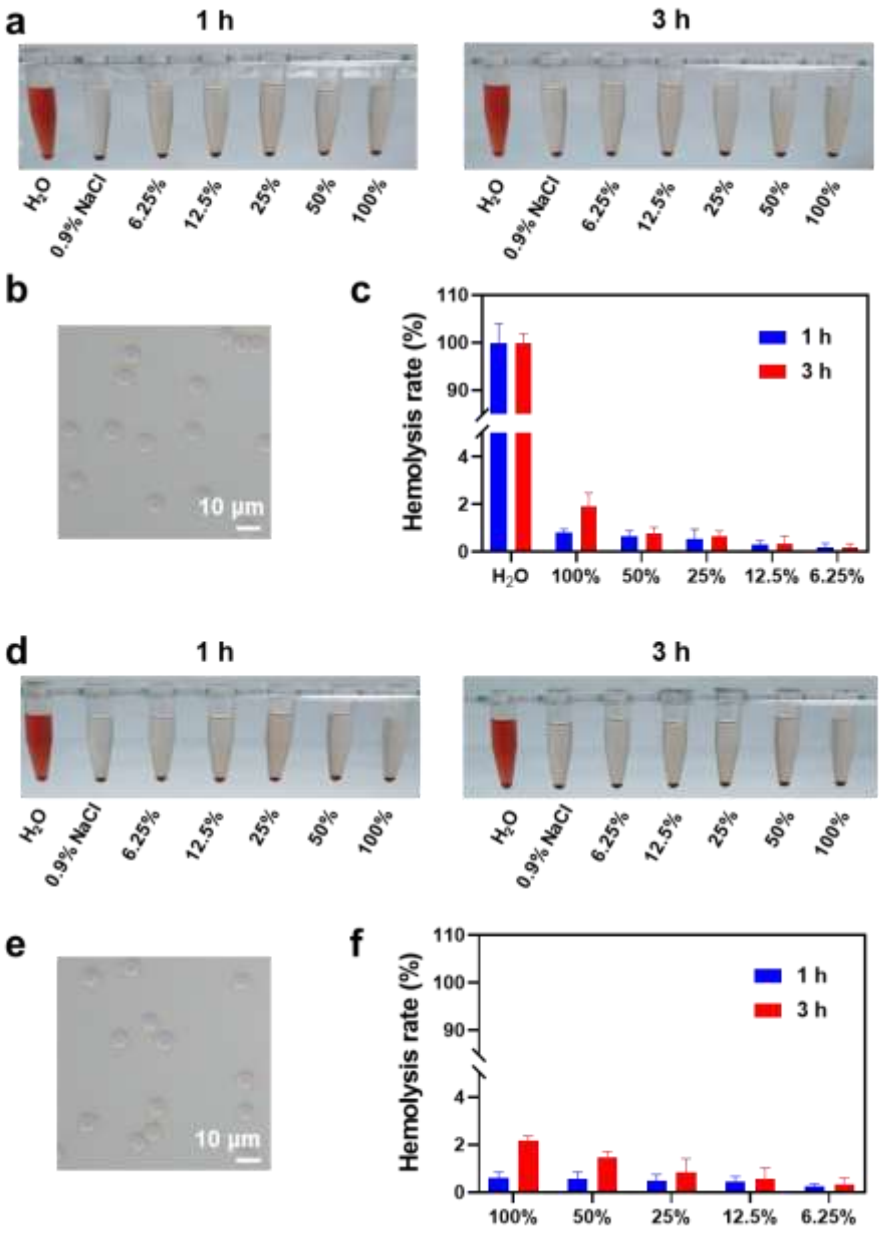

Figure S19. (a) Images showing the 2% red blood cell suspension incubated with 4aPEG-OPA/gelatin extracts for 1 h and 3 h, respectively. (b) The morphology of red blood cells incubated with 4aPEG-OPA/gelatin extract for 3 h. (c) Hemolysis rate of the 4aPEG-OPA/gelatin extracts after incubation with erythrocytes for 1 h and 3 h (mean ± SD, n = 5). (d) Images showing the 2% red blood cell suspension incubated with 4aPEG-OPA/4aPEG-SSNH$_2$ extracts for 1 h and 3 h, respectively. (e) The morphology of red blood cells incubated with 4aPEG-OPA/4aPEG-SSNH$_2$ extracts for 3 h. (f) Hemolysis rate of the 4aPEG-OPA/4aPEG-SSNH$_2$ extracts after incubation with red blood cells for 1 h and 3 h (mean ± SD, n = 5).

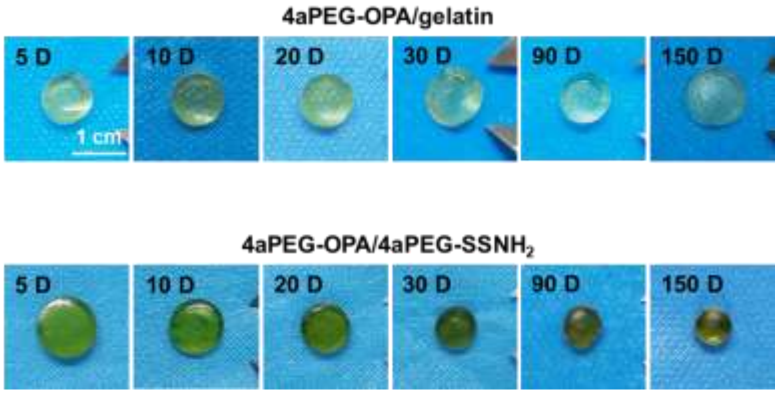

Figure S20. Remaining hydrogels taken out at different time points in rat subcutaneous degradation experiments.

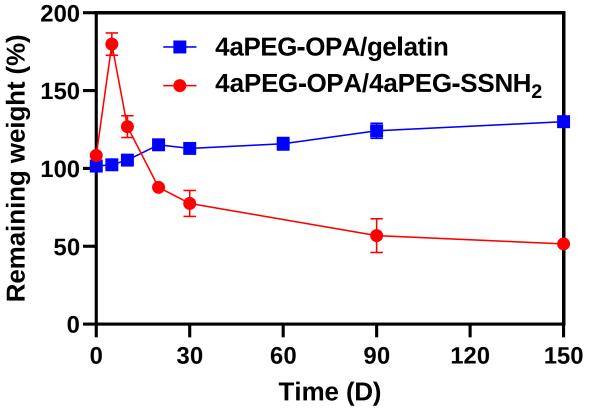

Figure S21. The weights of the remaining hydrogels taken out at different time points in the rat subcutaneous degradation experiment (n = 3).

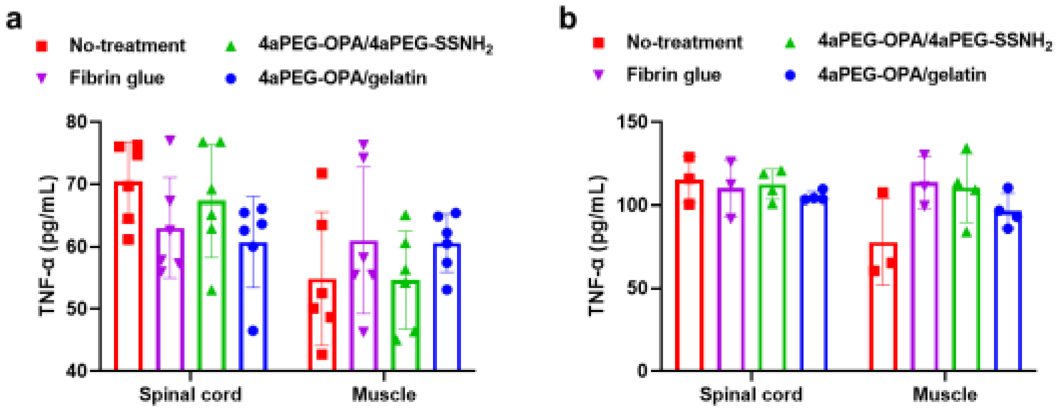

Figure S22. (a) TNF-α content of spinal cord and surface muscle for no-treatment, fibrin glue, 4aPEG-OPA/4aPEG-SSNH$_2$, and 4aPEG-OPA/gelatin groups at 1 week. (b) TNF-α content of spinal cord and surface muscle for different groups at 2 weeks.

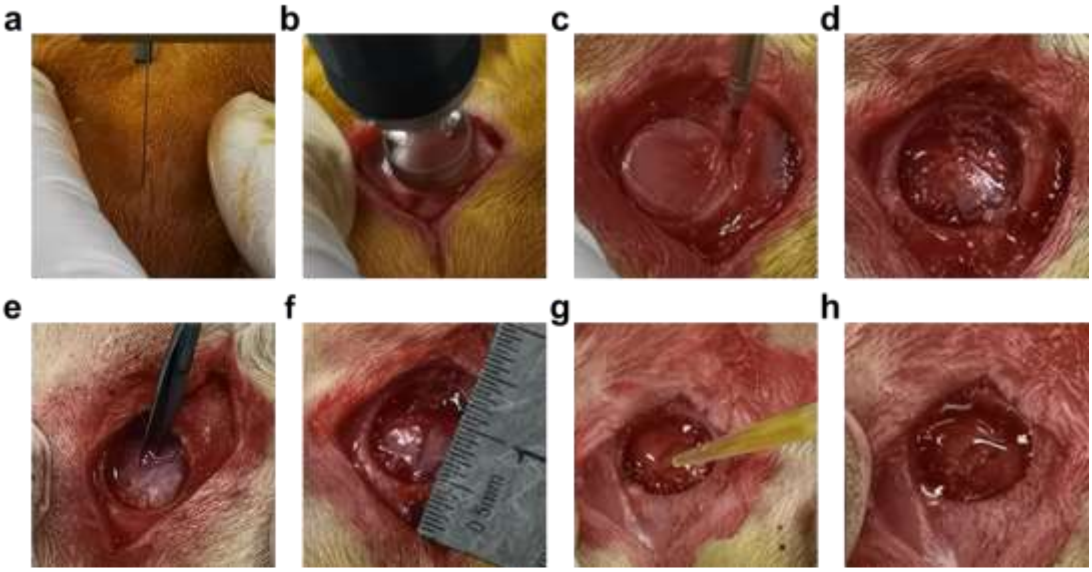

Figure S23. Surgical procedures for creation and sealing of cerebral dural defects in rabbits. (a) A skin incision was made on the top of rabbit's forehead after shave and disinfection. (b) A skull defect with diameter of 10 mm was prepared by using ring drill. (c) The skull was carefully stripped to avoid damage of dura mater. (d) The cerebral dura mater was exposed. (e) The dura mater and arachnoid were cut with microsurgical scissors. (f) The dural defect was 5 mm in length. (g) The precursor solution was added. (h) In situ formed hydrogel sealed the dural defect.

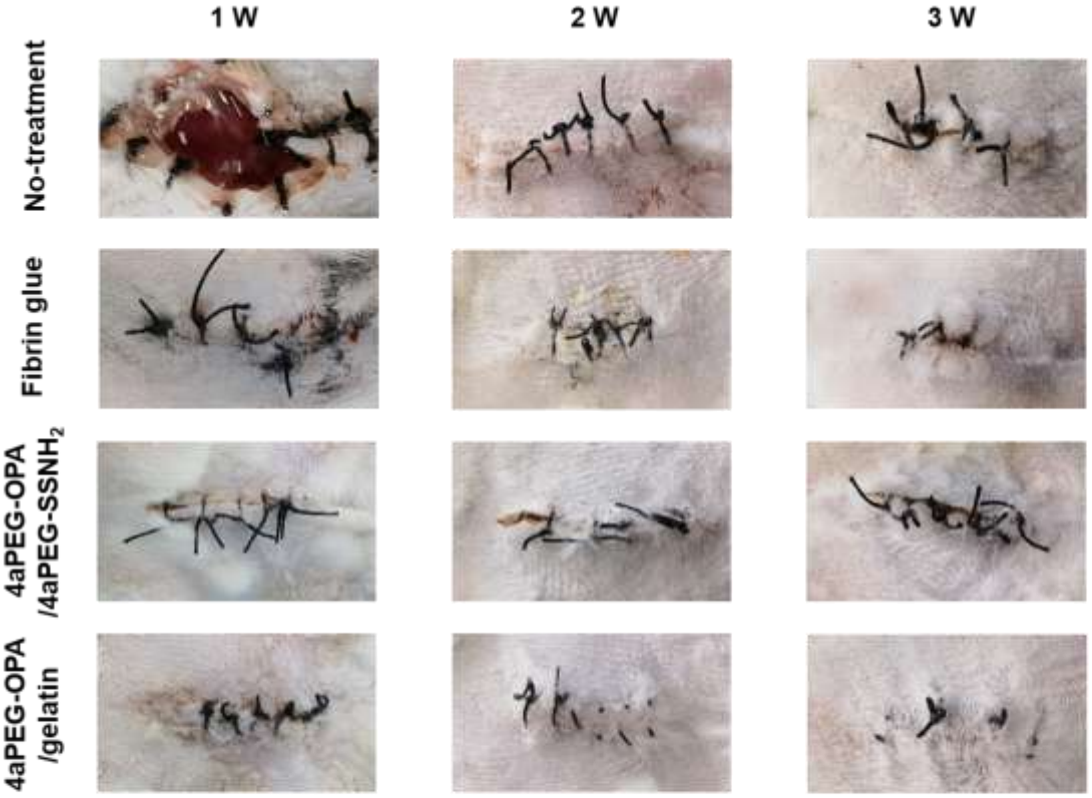

Figure S24. Visual inspection of surgical sites before dissecting the skin incisions for the no-treatment, fibrin glue, 4aPEG-OPA/4aPEG-SSNH$_2$, and 4aPEG-OPA/gelatin groups.

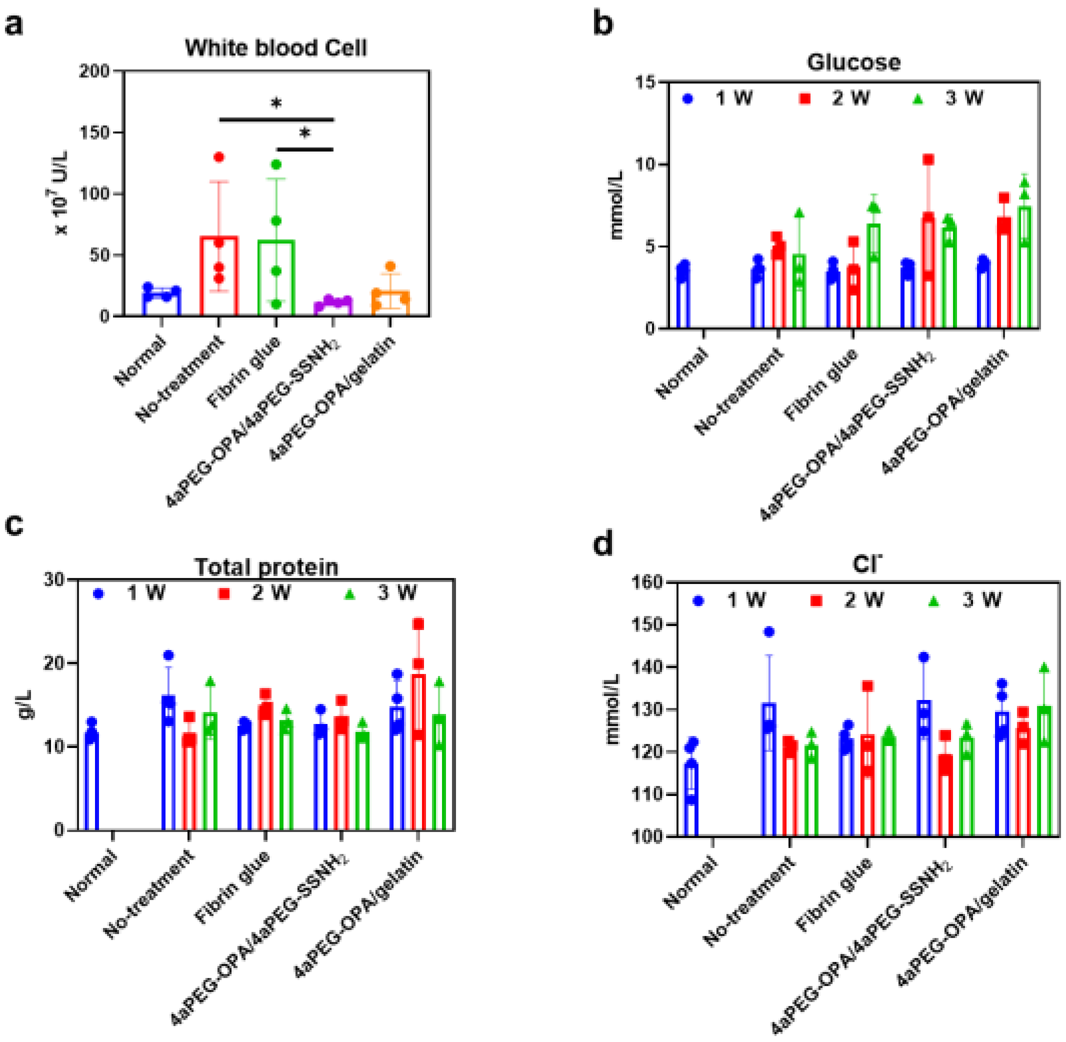

Figure S25. White blood cell count and contents of glucose, chloride, and total protein in the cerebrospinal fluid of rabbits for different groups at different time points (n = 3 ~ 4). *P < 0.05.

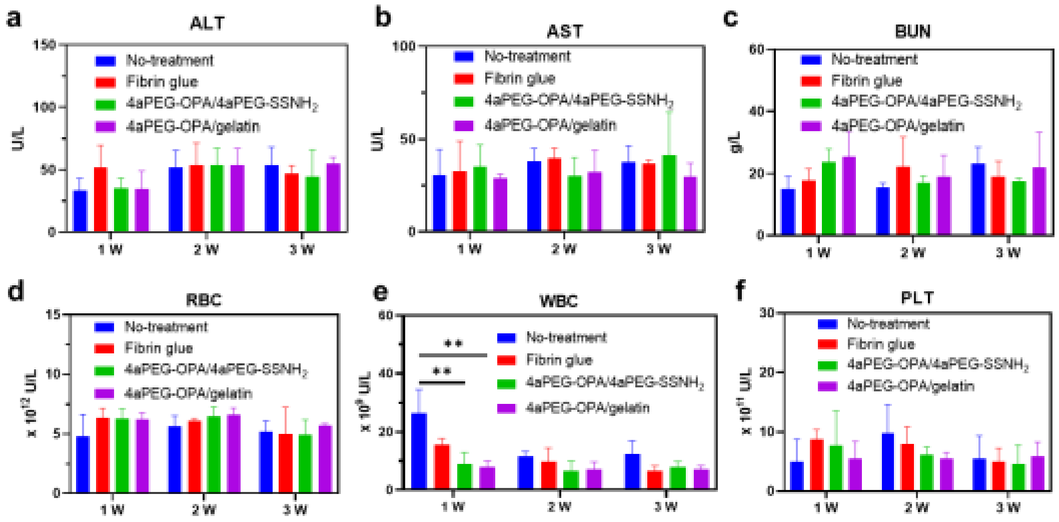

Figure S26. Blood routine and blood biochemical analysis after different treatments (no-treatment, fibrin glue, 4aPEG-OPA/4aPEG-SSNH$_2$, and 4aPEG-OPA/gelatin) of cerebral dural defect at 1 week, 2 weeks, and 3 weeks. ALT: alanine aminotransferase; AST: aspartate aminotransferase; BUN: blood urea nitrogen; RBC: red blood cell; WBC: white blood cell; PLT: platelet. The results are not significantly different between groups unless otherwise specified. **$P < 0.01$.

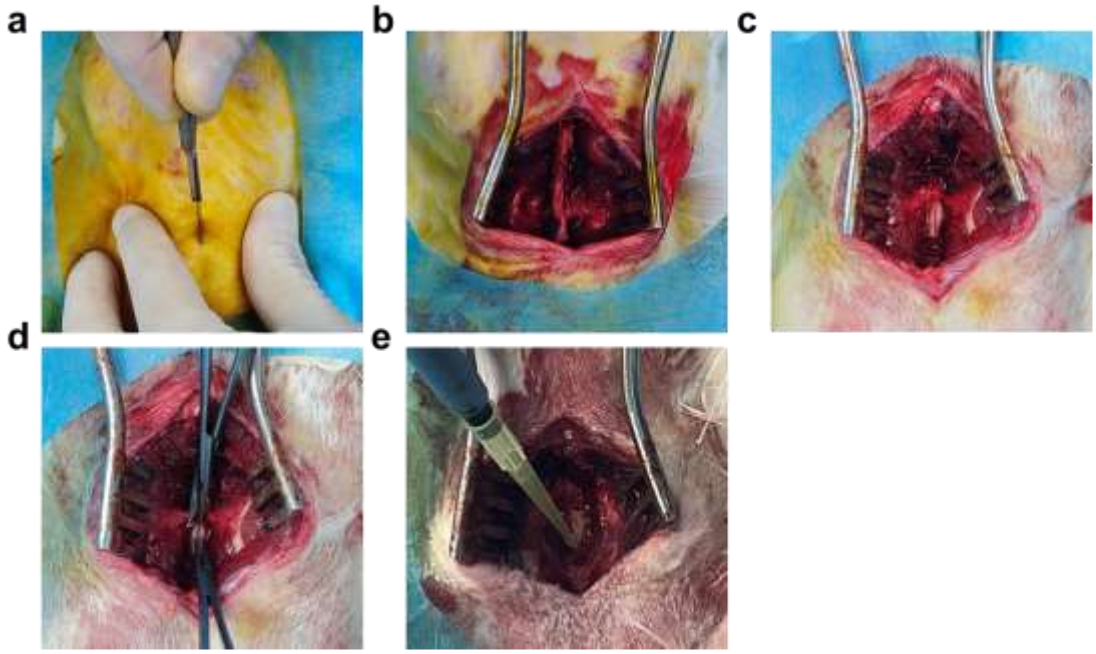

Figure S27. Surgical procedures for creating and sealing of lumbar dural defects in rabbits. (a) A skin incision was made over L4-L5 spinous process after disinfection. (b) Bilateral perispinous muscles were separated to fully expose the spinous process and lamina. (c) The lumbar dura mater was exposed after removing the lamina. (d) The dura mater and arachnoid were cut with microsurgical scissors. (e) In situ formed hydrogel sealed the dural defects after adding the precursor solution.

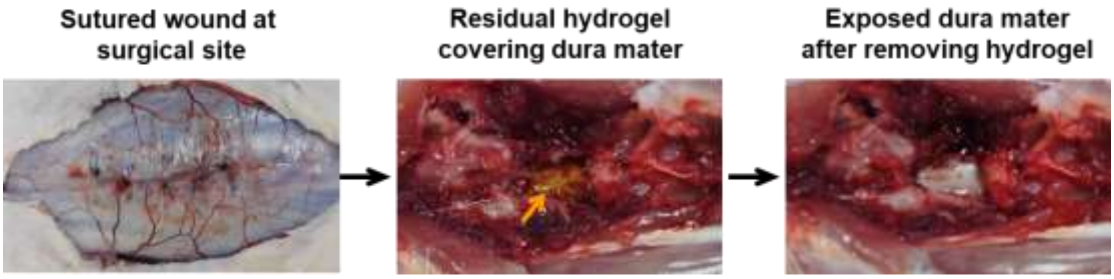

Figure S28. Images showing that the 4aPEG-OPA/gelatin hydrogel could seal the dural defect and prevent the postoperative adhesion in rabbit lumbar dura mater. Orange arrow represents the residual hydrogel.

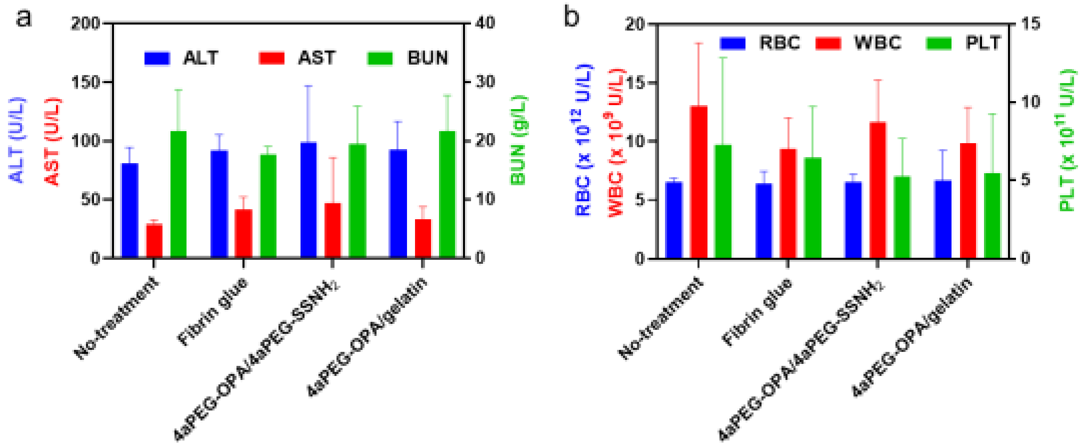

Figure S29. Blood routine and blood biochemical analysis after different treatments (no-treatment, fibrin glue, 4aPEG-OPA/4aPEG-SSNH$_2$, and 4aPEG-OPA/gelatin) of lumbar dural defect at 3 months. ALT: alanine aminotransferase; AST: aspartate aminotransferase; BUN: blood urea nitrogen; RBC: red blood cell; WBC: white blood cell; PLT: platelet. The results are not significantly different between groups unless otherwise specified.

Table S1. Comparison of compositions, adhesion mechanisms, properties, advantages and challenges of recently reported hydrogels for dural sealing.

Code	Building blocks	Gelation mechanism	Tissue adhesion mechanism	Burst pressure (cmH_2O)	Swelling ratio	Advantages	Challenges	Ref.
1	Tannic acid, pluronic F-127	Hydrogen bonding	Hydrogen bonding	39.7 ±4.5	/	/	Weak tissue adhesion	9
2	4aPEG-NHS, α-linolenic acid-modified gelatin	Amidation	Amidation	101.9 ±14.6	84 ±8%	/	Hydrolysis instability of NHS ester	10
3	4aPEG-NHS, 4aPEG-NH_2	Amidation	Amidation	94.3 ±2.7	538 ±68%	/	Hydrolysis instability of NHS ester; high swelling	11
4	4aPEG-NHS, α-CD and C10-modified gelatin	Amidation, hydrophobic interaction	Amidation	126.3 ±23.8	40 ±3%	Low swelling	Hydrolysis instability of NHS ester	12
5	Alginate, polyacrylamide, Ca^{2+}	Radical polymerization, ionic interaction	Amidation	79.0 ±11.6	521 ±30%	/	Involving radical initiator, EDC; high swelling	13
6	Pluronic F-127-bis-AA, HA-MA, acrylic acid-NHS	Photo-polymerization, micellar self-assembly	Amidation	229.2 ±18.6	-1% − 1%	Low swelling; high burst pressure	Involving photoinitiator and UV irradiation	14
7	4aPEG-OPA, gelatin	Phthalimidine bond	Phthalimidine bond	208.0 ±38.0	33.3 ±5.0%	Low swelling; high burst pressure; catalyst-free	/	This work

References

[1] Zhang Z, He C, Rong Y *et al*. A fast and versatile cross-linking strategy via o-phthalaldehyde condensation for mechanically strengthened and functional hydrogels. *Natl Sci Rev* 2021; 8: nwaa128.

[2] Feng M, Hu S, Qin W *et al*. Bioprinting of a blue light-cross-linked biodegradable hydrogel encapsulating amniotic mesenchymal stem cells for intrauterine adhesion prevention. *ACS Omega* 2021; 6: 23067-75.

[3] Mehdizadeh M, Weng H, Gyawali D *et al*. Injectable citrate-based mussel-inspired tissue bioadhesives with high wet strength for sutureless wound closure. *Biomaterials* 2012; 33: 7972-83.

[4] Jung O, Smeets R, Hartjen P *et al*. Improved in vitro test procedure for full assessment of the cytocompatibility of degradable magnesium based on iso 10993-5/-12. *Int J Mol Sci* 2019; 20: 255.

[5] Lewis KM, Sweet J, Wilson ST *et al*. Safety and efficacy of a novel, self-adhering dural substitute in a canine supratentorial durotomy model. *Neurosurgery* 2018; 82: 397-406.

[6] McGill F, Heyderman RS, Michael BD *et al*. The uk joint specialist societies guideline on the diagnosis and management of acute meningitis and meningococcal sepsis in immunocompetent adults. *J Infect* 2016; 72: 405-38.

[7] Puy V, Zmudka-Attier J, Capel C *et al*. Interactions between flow oscillations and biochemical parameters in the cerebrospinal fluid. *Front Aging Neurosci* 2016; 8: 154.

[8] Bosworth A, Bhatt K, Nance A *et al*. Elevated lactate levels in the cerebrospinal fluid associated with bacterial meningitis. *J Infect* 2019; 79: 389-99.

[9] Wang Y, Xie C, Wang P et al. An elastic gel consisting of natural polyphenol and pluronic for simultaneous dura sealing and treatment of spinal cord injury. *J Controlled Release* 2020; 323: 613-23.

[10] Mizuno Y, Taguchi T. Fish gelatin-based absorbable dural sealant with anti-inflammatory properties. *ACS Biomater Sci Eng* 2021; 7: 4991-8.

[11] Zhu T, Wang H, Jing Z *et al*. High efficacy of tetra-PEG hydrogel sealants for sutureless dural closure. *Bioact Mater* 2022; 8: 12-9.

[12] Komatsu H, Watanabe S, Ito S *et al*. Improved swelling property of tissue adhesive hydrogels based on alpha-cyclodextrin/decyl group-modified Alaska pollock gelatin inclusion complexes. *Macromol Biosci* 2023; 23: e2300097.

[13] Li J, Tian J, Li C *et al*. A hydrogel spinal dural patch with potential anti-inflammatory, pain relieving and antibacterial effects. *Bioact Mater* 2022; 14: 389-401.

[14] Bian S, Hao L, Qiu X *et al*. An injectable rapid-adhesion and anti-swelling adhesive hydrogel for hemostasis and wound sealing. *Adv Funct Mater* 2022; 32: 2207741.
